# Microarray Analysis of LTR Retrotransposon Silencing Identifies Hdac1 as a Regulator of Retrotransposon Expression in Mouse Embryonic Stem Cells

**DOI:** 10.1371/journal.pcbi.1002486

**Published:** 2012-04-26

**Authors:** Judith Reichmann, James H. Crichton, Monika J. Madej, Mary Taggart, Philippe Gautier, Jose Luis Garcia-Perez, Richard R. Meehan, Ian R. Adams

**Affiliations:** 1 MRC Human Genetics Unit, Institute of Genetics and Molecular Medicine, University of Edinburgh, Western General Hospital, Edinburgh, United Kingdom; 2 GENYO, Pfizer-University of Granada-Andalusian Government-Centre for Genomics and Oncological Research, Granada, Spain; Weizmann Institute of Science, Israel

## Abstract

Retrotransposons are highly prevalent in mammalian genomes due to their ability to amplify in pluripotent cells or developing germ cells. Host mechanisms that silence retrotransposons in germ cells and pluripotent cells are important for limiting the accumulation of the repetitive elements in the genome during evolution. However, although silencing of selected individual retrotransposons can be relatively well-studied, many mammalian retrotransposons are seldom analysed and their silencing in germ cells, pluripotent cells or somatic cells remains poorly understood. Here we show, and experimentally verify, that cryptic repetitive element probes present in Illumina and Affymetrix gene expression microarray platforms can accurately and sensitively monitor repetitive element expression data. This computational approach to genome-wide retrotransposon expression has allowed us to identify the histone deacetylase Hdac1 as a component of the retrotransposon silencing machinery in mouse embryonic stem cells, and to determine the retrotransposon targets of Hdac1 in these cells. We also identify retrotransposons that are targets of other retrotransposon silencing mechanisms such as DNA methylation, Eset-mediated histone modification, and Ring1B/Eed-containing polycomb repressive complexes in mouse embryonic stem cells. Furthermore, our computational analysis of retrotransposon silencing suggests that multiple silencing mechanisms are independently targeted to retrotransposons in embryonic stem cells, that different genomic copies of the same retrotransposon can be differentially sensitive to these silencing mechanisms, and helps define retrotransposon sequence elements that are targeted by silencing machineries. Thus repeat annotation of gene expression microarray data suggests that a complex interplay between silencing mechanisms represses retrotransposon loci in germ cells and embryonic stem cells.

## Author Summary

Repetitive DNA sequences make up almost half the mammalian genome. A large proportion of mammalian repetitive DNA sequences use RNA intermediates to amplify and insert themselves into new locations in the genome. Mammalian genomes contain hundreds of different types of these mutagenic retrotransposons, but the mechanisms that host cells use to silence most of these elements are poorly understood. Here we describe a computational approach to monitoring expression of hundreds of different retrotransposons in gene expression microarray datasets. This approach reveals new retrotransposon targets for silencing mechanisms such as DNA methylation, histone modification and polycomb repression in mouse embryonic stem cells, and identifies the histone deacetylase Hdac1 as a regulator of retrotransposons in this cell type. These computational predictions are verified experimentally by qRT-PCR in *Dnmt1^−/−^ Dnmt3a^−/−^ Dnmt3b^−/−^* embryonic stem cells, *Ring1B^−/−^* embryonic stem cells, and *Hdac1^−/−^* embryonic stem cells. We also use microarray analysis of retrotransposon expression to show that the pluripotency-associated *Tex19.1* gene has exquisite specificity for *MMERVK10C* elements in developing male germ cells. Importantly, our computational analysis also suggests that different genomic copies of individual retrotransposons can be differentially regulated, and helps identify the sequences in these retrotransposons that are being targeted by the host cell's silencing mechanisms.

## Introduction

Repetitive DNA sequences account for around forty percent of sequenced mammalian genomes [1], [2]. The most basic repetitive elements in mammalian genomes are tandem arrays of repeated monomeric DNA sequences. These simple repeats and satellite sequences have repeating units of around 1–5 bp and 100–500 bp respectively [3]. More complex classes of repetitive element include DNA transposons and retrotransposons, mobile genetic elements that are able to integrate into new sites in the genome. DNA transposons typically encode a transposase enzyme that catalyses the non-replicative mobilization of the DNA transposon through a cut and paste mechanism [4]. In contrast, retrotransposons mobilize using a replicative copy and paste mechanism that involves an RNA intermediate. However, this retrotransposition can occur by fundamentally different mechanisms depending on the structure of the retrotransposon [5], [6]. DNA transposons and retrotransposons account for ∼0.9% and ∼37% of the mouse genome respectively [2]. However, while DNA transposon activity appears to be extinct in the mouse genome, retrotransposons remain active [2]. Mouse retrotransposons include long interspersed elements (LINEs), short interspersed elements (*SINE*s), and long terminal repeat (LTR) retrotransposons [3]. Full-length class I LINEs are ∼7 kb long and encode two proteins that are required for the reverse-transcription of *LINE-1* RNA and its subsequent integration into new sites in the genome [7]. SINEs are derived from reverse-transcription of small cellular RNAs and utilise *LINE-1* proteins *in trans* to mediate retrotransposition [8]. LTR retrotransposons, also known as endogenous retroviruses (ERVs), either encode gag, *pol*, *pro* and sometimes also *env* genes, or use the retroviral genes encoded by other ERVs, to drive a retroviral life-cycle [2], [3], [9].

Retrotransposons have the potential to alter the genomic landscape and change gene expression when they amplify or integrate into new sites in the host genome, providing an important driving force for evolutionary change [10]. Although retrotransposition can occur in somatic cells [11], [12], repetitive elements need to amplify in germ cells, or their pluripotent precursors, in order to successfully propagate. The Repeatmasker database of repetitive elements [13] currently contains consensus sequences for 1221 different types of repetitive element, each of which is present in multiple copies in the mouse genome. These 1221 repetitive elements are organized into 16 different classes comprising a total of 45 families (see Figure S1 for a schematic overview of this organization). The repetitive element classes that contain the greatest number of different repetitive elements are LTR retrotransposons (471 elements), simple repeats (315 elements), DNA transposons (156 elements) and *LINE* retrotransposons (122 elements). Many of the repetitive elements that are present in the mammalian genome are poorly characterized, and it is often not clear whether different elements within each class or family are active at similar stages of germ cell or pluripotent cell development, or whether different elements are recognized and regulated by the same host defence mechanisms. Indeed the rich diversity of successful repetitive elements in the mammalian genome may indicate that different elements have evolved different strategies to evade recognition or suppression by host defence mechanisms.

The high mutational load associated with excessive amplification of repetitive elements in the developing germline is likely to be detrimental to the evolutionary success of the host organism. Much progress has been made in identifying and understanding the mechanisms that suppress the activity of repetitive elements in germ cells and pluripotent cells, particularly transcriptional repression of retrotransposon activity in mice [reviewed in 14]–[17]. Epigenetic modifications such as DNA methylation, histone methylation and histone deacetylation are all implicated in transcriptional silencing of retrotransposons. DNA methylation is required for transcriptional repression of intracisternal A particle (*IAP*) elements, a member of the ERVK family of LTR retrotransposons, in somatic cells and germ cells [18], [19]. Targeting DNA methylation to IAP elements during male fetal germ cell development requires the interaction between the piwi-piRNA pathway and DNA methyltransferase enzymes [reviewed in 15]–[17]. In pluripotent cells such as embryonic stem (ES) cells, mutations in all three catalytically active DNA methyltransferases greatly reduce the levels of DNA methylation in the genome [20], and these Dnmt1*^−/−^ Dnmt3a^−/−^ Dnmt3b^−/−^* triple knock out (Dnmt TKO) ES cells have increased expression of *IAP* retrotransposons [21], [22]. However, the increase in *IAP* expression in Dnmt TKO ES cells is relatively modest compared to somatic cells, and ES cells appear to rely more on the transcriptional co-repressor Kap1 to repress *IAP* elements [21]–[23]. Kap1 probably acts through recruitment of histone H3K9 methyltransferases, primarily Eset (also known as Setdb1 or Kmt1e), to deposit repressive histone modifications on *IAP chromatin* [22], [23]. Together Kap1 and Eset have been shown to target various ERV1, ERVK and ERVL LTR retrotransposons [22]–[24]. However, different silencing mechanisms are likely to be operating on retrotransposons that are not enriched for H3K9 methylation in mouse ES cells [16], [25]. Polycomb repressive complex (PRC)-mediated H3K27 trimethylation and Lsd1-dependent H3K4 demethylation are also implicated in transcriptional repression of LTR retrotransposons in mouse ES cells [26], [27], and histone deacetylation has been implicated in transcriptional silencing of newly-integrated *LINE-1* elements in undifferentiated human embryonal carcinoma (EC) cells [28]. Histone deacetylases, DNA methyltransferases, histone lysine methyltransferases and PRC proteins are all also implicated in transcriptional silencing of retroviral LTRs in human somatic cells [e.g. 29], [30], and some of the mechanisms operating to repress retrotransposon transcription in somatic cells may operate in pluripotent cells too. In addition to transcriptional silencing, retrotransposon activity is also regulated at post-transcriptional levels in germ cells and pluripotent cells through the activity of miRNAs and endogenous small interfering RNAs (endo-siRNAs) [31]–[33]. Other host factors, such as Apobec proteins [34] and the Trex1 endonuclease [35], have been shown to suppress retrotransposon activity post-transcriptionally in somatic cell types, and similar factors presumably also operate in pluripotent cells [36] and germ cells. Thus, multiple mechanisms probably combine to bring about effective silencing of different classes of retrotransposon in different cell types.

Although silencing of repetitive elements has been studied by qRT-PCR and Northern blotting of representative candidate elements in ES cells and in other cell types, few genome-wide studies of repetitive element expression have been performed to date [22], [23], [37]. Therefore it is often not clear how many different repetitive elements are being targeted by a specific silencing mechanism in any particular cell type. Given the antagonistic evolutionary relationship between retrotransposon expression and host silencing mechanisms, identifying repetitive elements that have escaped specific host silencing mechanisms may generate some insight into how these mechanisms are able to determine which regions of the genome or transcriptome to target. Microarrays are widely used for gene expression profiling, and a large volume of microarray gene expression data obtained under various experimental conditions has been deposited in freely-accessible repositories such as NCBI GEO [38]. Microarray analysis of gene expression has been able to identify some changes in repetitive element gene expression [e.g. 26], [39], but although a number of probes present on commercially available microarrays are identical to repetitive element sequences, few probes on these arrays are explicitly annotated as recognising repetitive elements.

The purpose of this study is to computationally extract information about genome-wide silencing of repetitive elements in germ cells and stem cells from microarray gene expression data. Using this approach we identify retrotransposons that are silenced by DNA methylation and various histone modifications in mouse embryonic stem cells. We also identify the histone deacetylase Hdac1 as a regulator of retrotransposons in mouse ES cells. Our results demonstrate that different silencing mechanisms can be independently recruited to retrotransposons in a modular manner, and that different genomic copies of individual retrotransposons can be differentially sensitive to loss of these silencing mechanisms. Lastly, we show that analysing the sequence variation between differentially regulated copies of individual retrotransposons can help identify sequences important for retrotransposon silencing.

## Results

### Identification of Repetitive Element Probes in the Illumina and Affymetrix Gene Expression Microarray Platforms

Previously, in a study designed to refine and improve the detection of gene expression changes in Illumina Mouse WG-6 Beadchip microarrays data, more than 4,000 probes in the Illumina Mouse WG-6 Beadchips were identified that map to regions of the mouse genome that are at least partially masked by Repeatmasker [40]. Although information from these probes was discarded from gene expression microarray data in that study in order to improve the analysis of the remaining single-copy probes [40], these repeat probes could potentially contain information about genome-wide repetitive element expression in microarray datasets. We therefore investigated how well different classes of repetitive element are represented in Illumina Beadarrays, and whether these probes could monitor repetitive element expression on a genome-wide level.

The Illumina Mouse WG-6 Beadchips each contain ∼46,000 probes. We identified ∼2,300 repetitive element probes in version 1.0, version 1.1 and version 2.0 of these arrays (Table 1) by comparing the genomic locations of the probes with the Repeatmasked regions of the mouse genome (see Materials and Methods). The proportion of repetitive element probes identified on the Illumina Beadchips in this analysis (∼5%) is around half that reported previously [40]. This difference appears to be a consequence of using stricter criteria to identify repetitive element probes in the current study. In each version of the Illumina Mouse WG-6 Beadchip analyzed, ∼1400 probes were in the correct orientation to detect sense repetitive element transcripts. Text files containing the repetitive element probe names and sequences identified in the Illumina Mouse WG-6 Beadchip are included online (Datasets S1, S2, S3). Of the 1221 different repetitive elements in the mouse genome annotated in the Repeatmasker database, ∼320 are represented by probes in the different versions of the Illumina Mouse WG-6 Beadchips (Table 2). Repetitive elements belonging to the *LINE* and *SINE* classes are well represented on these arrays, and repetitive elements belonging to the LTR retrotransposon and DNA transposon classes are reasonably represented (Table 2). Simple repeats and satellite repeats are also present but less well represented on the Illumina Mouse WG-6 Beadchips (Table 2). Thus Illumina Mouse WG-6 Beadchips have a good coverage of probes for monitoring transposon and retrotransposon expression during genome-wide transcriptional profiling.

**Table 1:** Number of probes matching repetitive elements in mouse gene expression microarray platforms.

	Illumina			Affymetrix	
	WG6 v1.0	WG6 v1.1	WG6 v2.0	U74Av2	430 2.0
**All probes**	46,005	46,632	45,281	197,993	496,468
**Probes matching repetitive elements (non-complementary)**	899	912	867	2,636	19,870
**Probes matching repetitive elements (complementary)**	1,397	1,425	1,438	4,239	26,124

**Table 2:** Number of different repetitive elements represented by complementary probes in mouse gene expression microarray platforms.

	Mouse Genome	Illumina			Affymetrix	
	mm9 assembly	WG-6 v1.0	WG-6 v1.1	WG-6 v2.0	U74Av2	430 2.0
**LINE**	122 elements	70 elements	71 elements	66 elements	62 elements	97 elements
	1.3 million loci	351 probes	358 probes	321 probes	631 probes	4635 probes
**SINE**	41 elements	30 elements	30 elements	32 elements	32 elements	37 elements
	2.1 million loci	473 probes	486 probes	558 probes	1465 probes	11650 probes
**LTR**	471 elements	153 elements	155 elements	153 elements	107 elements	291 elements
	1.2 million loci	393 probes	396 probes	372 probes	1362 probes	7293 probes
**DNA**	156 elements	42 elements	43 elements	40 elements	36 elements	88 elements
	0.2 million loci	69 probes	71 probes	58 probes	229 probes	1329 probes
**Satellite**	8 elements	2 elements	2 elements	2 elements	2 elements	2 elements
	0.01 million loci	55 probes	54 probes	61 probes	266 probes	463 probes
**Simple**	315 elements	8 elements	3 elements	9 elements	26 elements	47 elements
	1.5 million loci	9 probes	9 probes	12 probes	67 probes	168 probes
**Other**	108 elements	13 elements	14 elements	13 elements	15 elements	32 elements
	0.6 million loci	47 probes	51 probes	56 probes	219 probes	586 probes
**Total**	1,221 elements	318 elements	323 elements	315 elements	280 elements	594 elements
	6.9 million loci	1397 probes	1425 probes	1438 probes	4239 probes	26124 probes

Mouse genome data is derived from Repeatmasker annotation of the mm9 assembly of the sequenced genome downloaded from the UCSC genome browser [62]. The number of elements within each repetitive element class that are represented in the mouse genome and in the different microarray platforms is indicated. The number of genomic loci or microarray probes corresponding to each repetitive element class is also shown.

We applied the same rationale to identify repetitive element probes present in the Affymetrix Murine Genome U74Av2 and Mouse Expression 430 2.0 GeneChips (Table 1). The Murine Genome U74Av2 and Mouse Expression 430 2.0 GeneChips contain ∼4,200 and ∼26,000 probes respectively that are in the correct orientation to detect sense transcripts from repetitive elements. Text files containing the repetitive element probe names and sequences identified in the Affymetrix Gene Expression GeneChips are included online (Datasets S4, S5). Like the Illumina Mouse WG-6 Beadchip arrays, the Affymetrix arrays also have good representation of repetitive elements belonging to *LINE* and *SINE* classes, and the Affymetrix Mouse Expression 430 2.0 GeneChip also has good coverage of LTR retrotransposons and DNA transposons (Table 2). The Affymetrix Murine Genome U74Av2 GeneChip has reasonable coverage of repetitive elements within the LTR retrotransposon and DNA transposon classes (Table 2). Thus Affymetrix Gene Expression GeneChips also contain a wide range probes that can be used to monitor transposon and retrotransposon expression.

### Computational Analysis of Repetitive Element Expression in *Tex19.1^−/−^* Testes from Microarray Gene Expression Profiles

**Figure 1:**
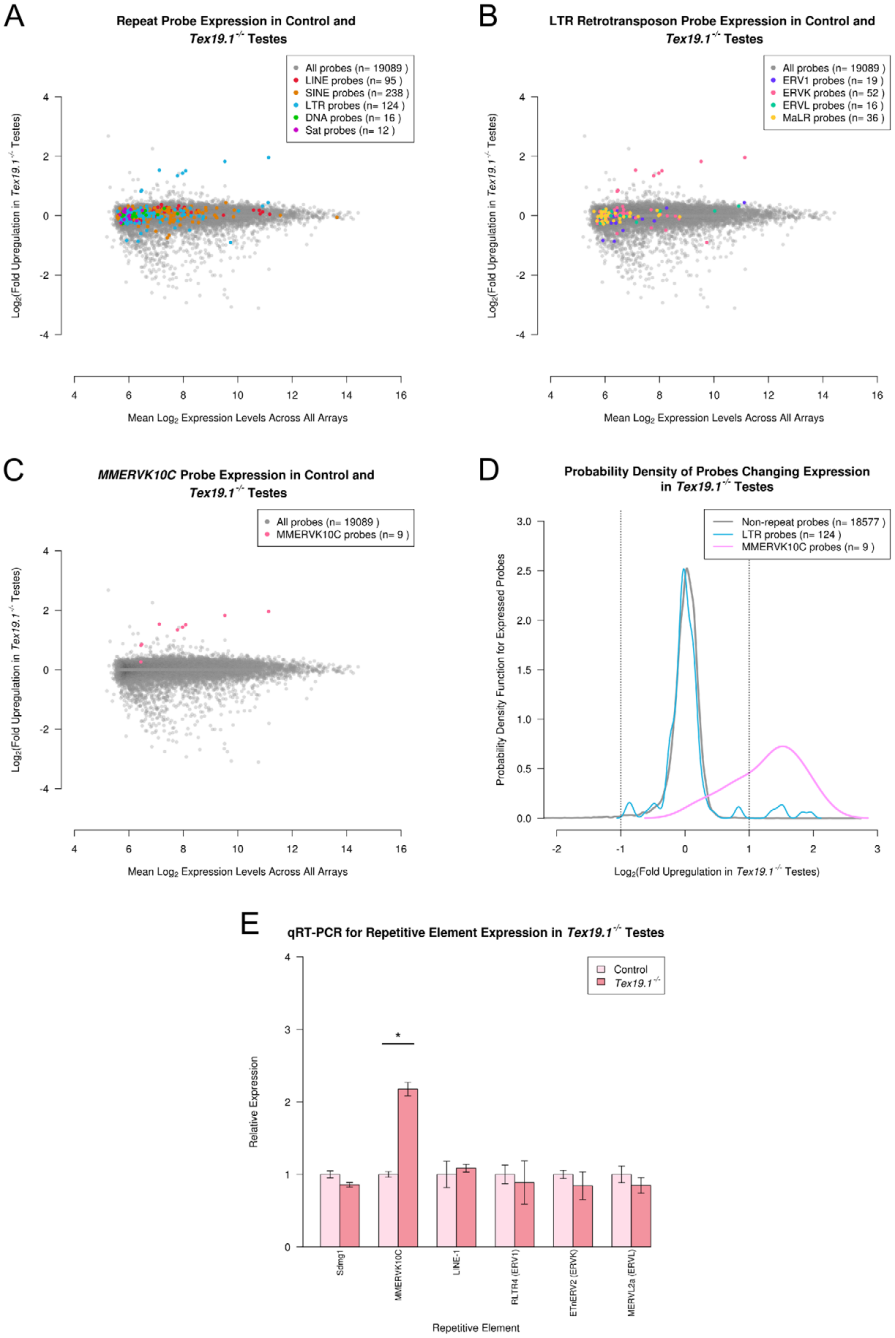
Genome-wide repetitive element expression in *Tex19.1^−/−^* testes. (A–C) MA-plots showing the mean expression level for each expressed probe in the *Tex19.1* testis Illumina Beadarray data plotted against the fold upregulation of that probe in *Tex19.1^−/−^* testes. Probes for repeat families (A), classes of LTR retrotransposons (B), and the *MMERVK10C* element (C) are colour-coded in each plot according to the legend. Note the group of six *MMERVK10C* ERVK LTR retrotransposon probes upregulated in *Tex19.1^−/−^* testes. (D) Plot showing the behaviour of the entire *MMERVK10C* probe population in *Tex19.1^−/−^* testes. Vertical lines indicate a 2 fold change. (E) qRT-PCR verification of *MMERVK10C* upregulation in C57BL/6 *Tex19.1^−/−^* testes. Expression levels for each repetitive element (mean ± standard error for three animals) were normalized to *β-Actin* and expressed relative to littermate controls. Representative LTR retrotransposons belonging to ERV1, ERVK and ERVL classes do not change expression in *Tex19.1^−/−^* testes. *Sdmg1* is a single-copy control gene for Sertoli cell expression to verify normalization between animals. MMERVK10C env.c and LINE1 ORF2 primer sets (Figure S2) were used to assess *MMERVK10C* and *LINE-1* expression. Asterisk indicates a statistically significant difference (p<0.05).

We had previously identified upregulation of the *MMERVK10C* (ERVK family) LTR retrotransposon in mouse germ cells lacking the pluripotency-associated *Tex19.1^−/−^* gene by analysing individual probe sequences upregulated in Illumina Beadchip microarray data [39]. In order to test whether any additional retrotransposons might be targets for *Tex19.1* in developing male germ cells we used the repeat probes in the Illumina Mouse WG-6 v2.0 Beadchip to assess genome-wide repetitive element expression in *Tex19.1^−/−^* testis microarray data. As *Tex19.1^−/−^* male mice have defects in progression through meiosis that perturb the normal cellular composition of the testis, gene expression profiling was performed on 16 dpp prepubertal testes undergoing the first wave of spermatogenesis where defects in meiosis are first becoming apparent [39]. In addition, the *Tex19.1* mutation was backcrossed onto an inbred C57BL/6 genetic background in order to minimize genetic variation between the animals used for this microarray analysis. 19,089 probes on the Illumina Beadarray were expressed in 16 dpp testes in this experiment (Figure 1A), with most showing no significant change in expression in *Tex19.1^−/−^* testes. The expression levels of 158 probes (0.8%) are downregulated at least 2 fold in *Tex19.1^−/−^* testes at a significance level of p<0.01. However, the apparent downregulation of many of these probes may be a consequence of the delay in meiotic progression that is becoming evident in *Tex19.1^−/−^* testes at 16 dpp [39]. On the other hand, 10 probes (0.05%) are upregulated at least 2 fold in *Tex19.1^−/−^* testes at p<0.01.

In general the repetitive element probes behaved similarly to other probes on the array (Figure 1A). 512 (2.7%) of the 19,089 probes expressed in 16 dpp testes are repeat probes. These 512 repeat probes represent 173 different repetitive elements. LTR retrotransposon, *LINE*, *SINE*, DNA transposon, and satellite transcripts were all expressed similarly in *Tex19.1^−/−^* and control testes (Figure 1A). However, 6 repeat probes belonging to the LTR retrotransposon class appear to be behaving as outliers from the total probe population (Figure 1A). These outlying probes are upregulated 2–4 fold in *Tex19.1^−/−^* testes, and all belong to the ERVK family of LTR retrotransposons (Figure 1B). All of these 6 probes are complementary to the *MMERVK10C* repetitive element (Figure 1C). Indeed, although the 124 LTR retrotransposon probes that are expressed in this dataset do not behave differently from the 18,577 non-repeat probes (Figure 1D, Wilcoxon rank sum test p = 0.5), the 9 *MMERVK10C* probes expressed in this dataset represent a distinct population from the non-repeat probes (Figure 1D, Wilcoxon rank sum test, p<0.0001). The *MMERVK10C* probes also appear to be behaving differently from other LTR retrotransposon and ERVK retrotransposon probes in this dataset (Wilcoxon rank sum tests, p<0.0001). Only four non-repeat probes are upregulated in *Tex19.1^−/−^* testes, and none of these probes map close to *MMERVK10C* loci in the reference genome, suggesting that the upregulation of *MMERVK10C* elements in *Tex19.1^−/−^* testes is likely to be caused by loss of a *trans*-acting retrotransposon silencing mechanism rather than changes in non-repetitive gene expression affecting the local chromatin structure and influencing expression of nearby retrotransposon sequences.

The unique behaviour of *MMERVK10C* repeat probes in the microarray data was confirmed by identifying probes whose expression changed at least 2 fold (p<0.01) in *Tex19.1^−/−^* testes relative to control testes. 6 (1.2%) of the 512 repeat probes change expression at least 2 fold (p<0.01) in *Tex19.1^−/−^* testes, and all 6 of these repeat probes are derived from *MMERVK10C-int* LTR retrotransposon sequences. We confirmed that each of these *MMERVK10C* probe sequences matches multiple genomic loci (≥48/50 nt identity) by BLAT suggesting that each probe is able to detect expression from multiple genomic copies of the *MMERVK10C* LTR retrotransposon (data not shown). Furthermore, we also confirmed that the non-complementary repeat probes recognizing antisense repetitive element transcripts did not show any significant change in expression in *Tex19.1^−/−^* testes (data not shown). Thus repeat-annotation of the *Tex19.1^−/−^* Illumina Beadchip data suggests that expression of *MMERVK10C* retrotransposons is significantly and specifically upregulated in *Tex19.1^−/−^* testes. The systematic annotation and analysis of the C57BL/6 *Tex19.1^−/−^* testis microarray data presented here is consistent with our previous findings that *MMERVK10C* elements are upregulated in *Tex19.1^−/−^* testes from a mixed (129/Ola×CD1) genetic background [39], but importantly also extends the range and variety of repetitive elements analysed in these animals. Intriguingly, MMERVK10C remains the only repetitive element among the 173 elements represented in this dataset whose expression changes by more than 2 fold in the absence of *Tex19.1*.

### Retrotransposon Derepression in *Tex19.1^−/−^* Testes Is Restricted to *MMERVK10C* Elements

Our computational analysis of *Tex19.1^−/−^* testis microarray data suggests that repetitive element misexpression in *Tex19.1^−/−^* testes is largely restricted to upregulation of *MMERVK10C* elements (Figure 1A–D). We verified the upregulation of *MMERVK10C* elements in an independent group of C57BL/6 *Tex19.1^−/−^* testes by qRT-PCR (Figure 1E). The ∼2 fold qRT-PCR upregulation of *MMERVK10C* elements in C57BL/6 *Tex19.1^−/−^* testes is similar to the ∼4 fold qRT-PCR upregulation of this element reported previously using animals on a mixed genetic background [39]. The slightly lower level of upregulation of MMERVK10C seen in C57BL/6 animals may be caused by differences in the rate of testis development between these genetic backgrounds. In order to investigate the apparent specificity of the *MMERVK10C* upregulation evident in the microarray analysis we tested expression of *LINE-1* and some representative ERV1, ERVK and ERVL LTR retrotransposon sequences in *Tex19.1^−/−^* testes by qRT-PCR. qRT-PCR for *LINE-1* retrotransposons (Figure 1E) confirmed the repeat-annotation analysis suggesting that these elements do not change expression in *Tex19.1^−/−^* testes (Figure 1A–D). Furthermore, *RLTR4*, *ETnERV2* and *MERVL2a* LTR retrotransposons representing the ERV1, ERVK and ERVL families of LTR retrotransposons also do not change expression in *Tex19.1^−/−^* testes in either the Illumina Beadarray data (Figure 1A–D) or by qRT-PCR (Figure 1E). Thus *MMERVK10C* elements appear to be behaving differently from other LTR retrotransposons in *Tex19.1^−/−^* testes.

**Figure 2:**
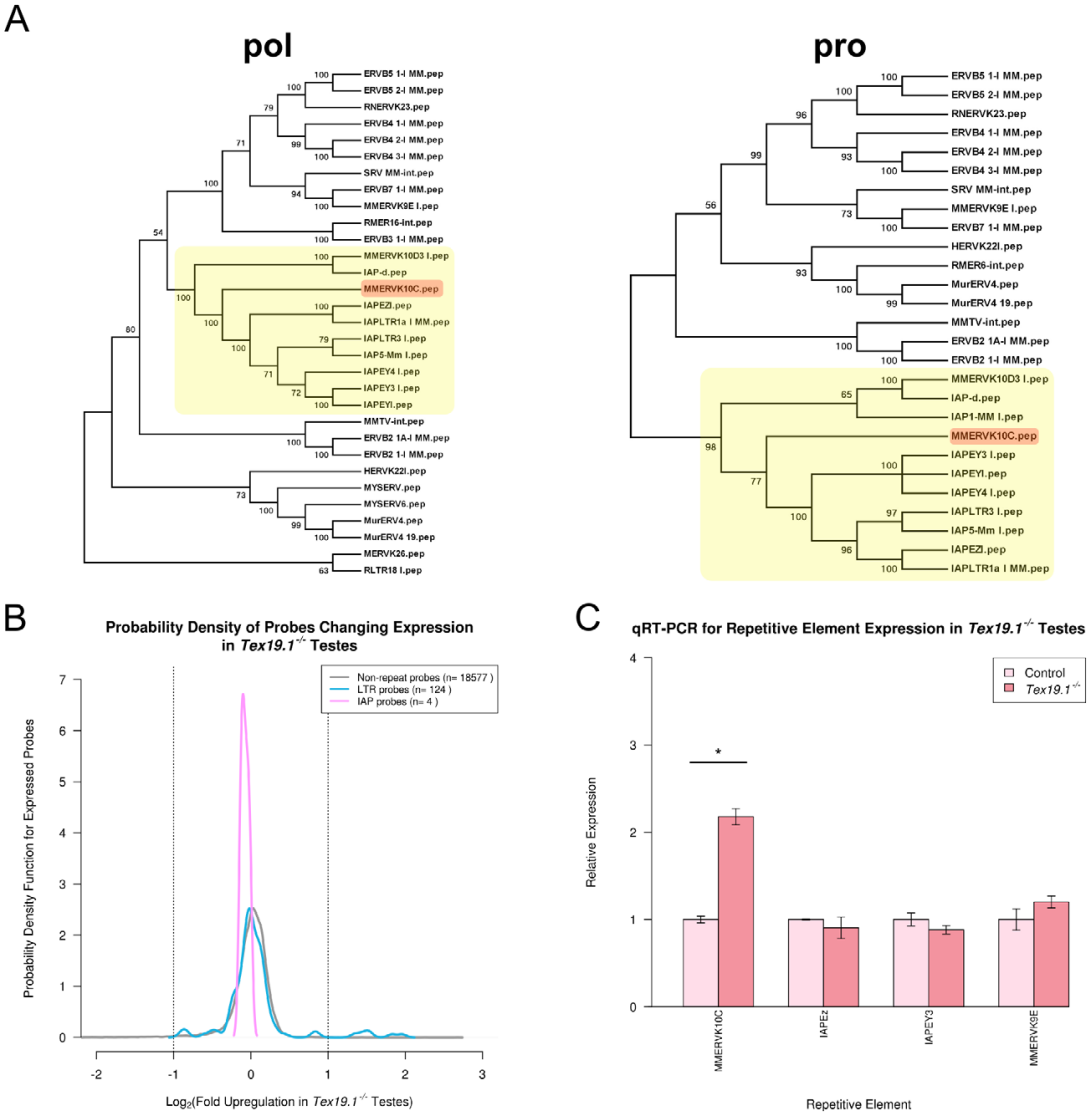
Closely related retrotransposons are differentially sensitive to loss of *Tex19.1*. (A) Phylogeny of mouse retrotransposon pol and pro proteins. *MMERVK10C* sequences are highlighted in red. The *MMERVK10C* sequences lie within a cluster of *IAP*-type sequences (yellow). (B) Plot showing the likelihood of *IAP* probes changing expression in the *Tex19.1^−/−^* microarray dataset. (C) qRT-PCR for retrotransposons closely related to *MMERVK10C* in *Tex19.1^−/−^* knockout and littermate control testes at 16 dpp. Expression levels for each repetitive element (mean ± standard error for three animals) were normalized to *β-Actin* and expressed relative to littermate controls. MMERVK10C env.c and IAP primer sets (Figure S2) were used to assess *MMERVK10C* and *IAPEz* expression. Asterisk indicates a statistically significant difference (p<*0.05*).

The Illumina Beadarrays used to profile gene expression in the *Tex19.1^−/−^* testes contain probes representing around a third of the LTR retrotransposons present in the mouse genome. Therefore although the computational and experimental data both suggest that *MMERVK10C* elements respond differently from other retrotransposons in the genome to the loss of *Tex19.1*, we investigated whether LTR retrotransposons that were closely related to *MMERVK10C* might also be upregulated in *Tex19.1^−/−^* testes. We used *MMERVK10C pol* and *pro* protein sequences to identify repetitive elements closely related to *MMERVK10C* (Figure 2A). *MMERVK10C* appears to be most closely related to *IAP* elements, with the *pol* protein sequences of *MMERVK10C*, *IAPEz* and *IAPEY3* all having around 75% similarity to each other. Although there are numerous *IAP* probes in the Illumina Beadarrays, these probes do not appear to be changing in *Tex19.1^−/−^* testes (Figure 2B). Furthermore we tested expression of *IAPEz* and *IAPEY3* elements in *Tex19.1^−/−^* testes by qRT-PCR (Figure 2C) and found that, as suggested by computational analysis of the microarray data, expression of these elements is not changing in *Tex19.1^−/−^* testes. We also tested expression of the *MMERVK9E* retrotransposon that is related to *MMERVK10C* but not represented on the Illumina Beadarrays. *MMERVK9E* has around 65% similarity to *MMERVK10C* across the *pol* protein sequence, but is not part of the cluster of *IAP* elements evident in the *MMERVK10C* phylogeny (Figure 2A). However, qRT-PCR data shows that *MMERVK9E* elements do not change expression in *Tex19.1^−/−^* testes either (Figure 2C). Thus retrotransposon derepression in *Tex19.1^−/−^* testes appears to be intriguingly restricted to *MMERVK10C* elements.

### Different Transcriptional Silencing Mechanisms Have Distinct Effects on Genome-Wide Repression of Repetitive Elements

Our data on *Tex19.1^−/−^* testes suggests that only a small number of retrotransposon RNAs are sensitive to loss of *Tex19.1* in germ cells. We therefore next investigated whether loss of well established retrotransposon silencing mechanisms had more extensive effects on genome-wide repression of retrotransposons using ES cells as a model. We computationally analysed repetitive element expression in previously published gene expression microarray datasets from Dnmt TKO ES cells carrying mutations in all three catalytically active DNA methyltransferases [41], and from ES cells transiently transfected with shRNAs to knock-down the histone H3K9 methyltransferase Eset [42]. Although the Dnmt TKO and *Eset^shRNA^* ES cell gene expression profiles were performed on Affymetrix and Illumina platforms respectively, and may therefore have some differences in coverage of individual retrotransposons or sensitivity of detection limits, different classes of repetitive elements are similarly represented on these platforms (Table 2) and some genome-wide comparisons will still be informative. We also included data from Affymetrix gene expression profiling of ES cells carrying mutations in the Hdac1 histone deacetylase enzyme [43] in this analysis. Although the HDAC family of histone deacetylases are implicated in retrotransposon silencing by virtue of being targets of trichostatin A [28], [44], [45], the role and retrotransposon targets of the different HDAC histone deacetylases has not yet been defined. Genome-wide analysis of retrotransposon silencing in Dnmt TKO, *Eset^shRNA^* and *Hdac1^−/−^* ES cells could therefore uncover new or additional retrotransposon targets for these mechanisms in ES cells.

**Figure 3:**
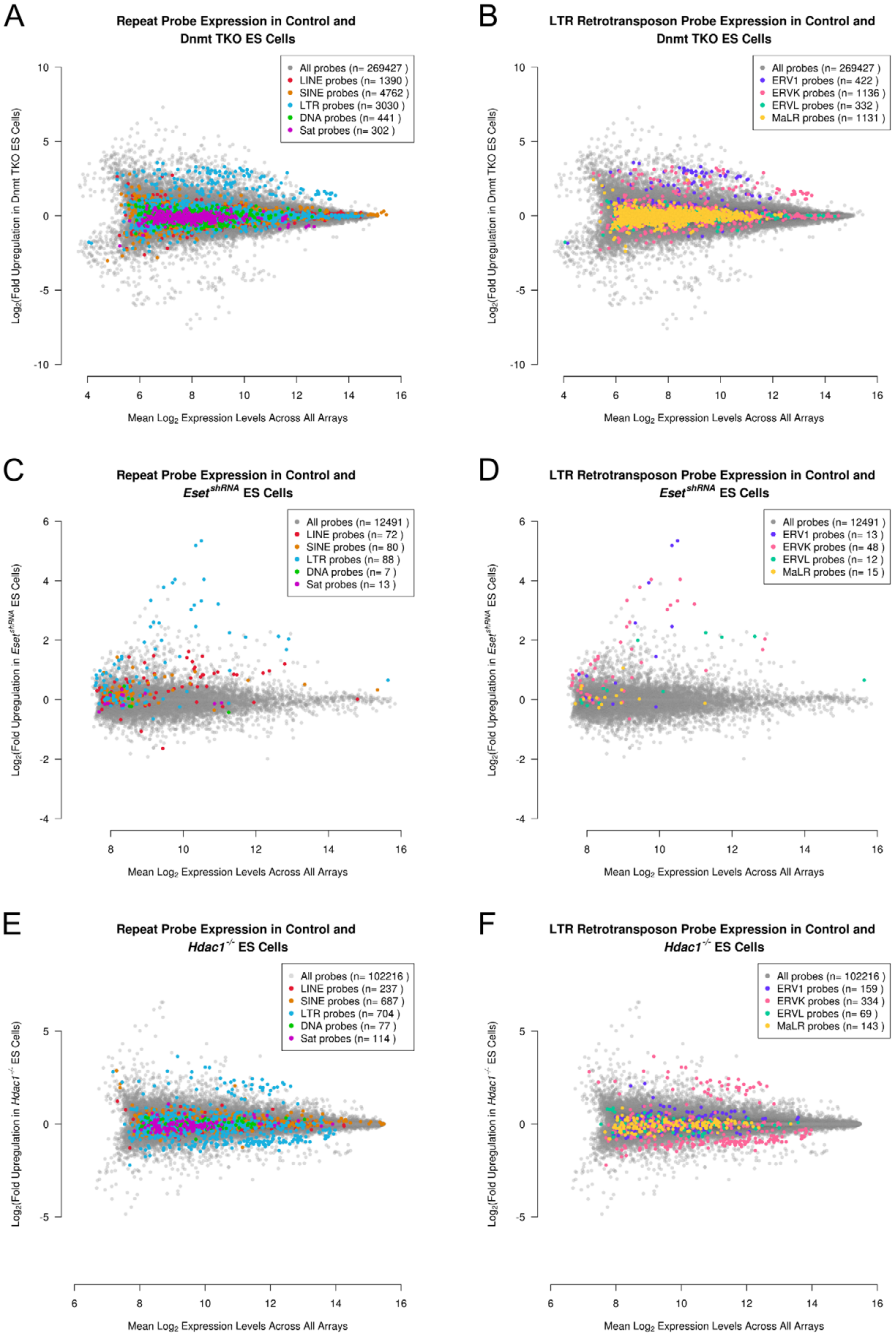
Different transcriptional silencing mechanisms have distinct effects on genome-wide repression of repetitive elements. (A, B) MA-plots for *Dnmt1^−/−^ Dnmt3A^−/−^ Dnmt3B^−/−^* triple knockout (Dnmt TKO) ES cell Affymetrix Gene Expression data. The mean expression level for each expressed probe is plotted against the fold upregulation of that probe in Dnmt TKO ES cells. Probes for repeat families (A), and classes of LTR retrotransposons (B) are colour-coded in each plot according to the legend. A group of ERV1 and ERVK LTR retrotransposons can be seen to be upregulated relative to the total probe population in the Dnmt TKO ES cells. (C, D) MA-plots for *Eset^shRNA^* ES cell Illumina Beadchip data with probes for repeat families (C), and classes of LTR retrotransposons (D) colour-coded according to the legend. Probes for different ERV1, ERVK and ERVL LTR retrotransposon families are strongly upregulated, and multiple *LINE-1* probes are modestly upregulated, in *Eset^shRNA^* ES cells. (E, F) MA-plots for *Hdac1^−/−^* ES cell Affymetrix Gene Expression data with probes for repeat families (E), and classes of LTR retrotransposons (F) colour-coded according to the legend. One group of ERVK LTR retrotransposon probes is upregulated in *Hdac1^−/−^* ES cells, another group is downregulated.

Repeat-annotation of Dnmt TKO, *Eset^shRNA^* and *Hdac1^−/−^* ES cells (Figure 3) confirmed that LTR retrotransposons are upregulated in all of these mutant ES cells. Interestingly, although individual retrotransposon sequences could be selected that show upregulation in each of these mutant ES cell lines, the genome-wide overview of retrotransposon behaviour shows striking differences in retrotransposon behaviour between mutant ES lines (Figure 3A, 3C, 3E). Dnmt TKO ES cells appear to modestly upregulate a number of LTR retrotransposon probes around 2–8 fold, which behave similarly to the upregulated non-repeat probes in the array, but other classes of repeat probe do not appear to change (Figure 3A). The upregulated group of LTR retrotransposon probes in Dnmt TKO ES cells is primarily composed of ERV1 and ERVK classes of LTR retrotransposon (Figure 3B). In contrast *Eset^shRNA^* ES cells appear to strongly upregulate most LTR retrotransposon probes in the array, and these upregulated LTR retrotransposon probes appear to be responding more strongly to loss of *Eset* than the upregulated non-repeat probes in the dataset (Figure 3C). The range of LTR retrotransposon probes upregulated in *Eset^shRNA^* ES cells is more expansive than in Dnmt TKO ES cells with probes belonging to ERV1, ERVK and ERVL classes all being upregulated (Figure 3D). Furthermore, *Eset^shRNA^* ES cells appear to modestly upregulate *LINE-1* probes (Figure 3C), a group of retrotransposons that does not strongly change expression in Dnmt TKO ES cells (Figure 3A). Thus *Eset* appears to have a stronger and more widespread role in repressing retrotransposons in ES cells than DNA methylation. Interestingly, *Hdac1* also plays a role in repressing retrotransposons in ES cells (Figure 3E). However the role of *Hdac1* appears to be distinct from the roles of DNA methylation and Eset histone methyltransferase. *Hdac1^−/−^* ES cells upregulate one group of LTR retrotransposon probes 4–8 fold, a relatively strong upregulation compared to non-repeat probes in the dataset, and downregulate a second large group of LTR retrotransposon probes around 2–4 fold (Figure 3E). The upregulated and downregulated groups of LTR retrotransposon probes are both primarily composed of ERVK class LTR retrotransposons (Figure 3F, pink dots), and these changes in ERVK probe expression are comparable in magnitude to the changes in non-repetitive gene expression that occur in *Hdac1^−/−^* ES cells (Figure 3F, grey dots, [43]). The observation that LTR retrotransposon expression is altered in Hdac1*^−/−^* ES cells is consistent with data showing that human *HDAC1* can silence avian retroviral LTR reporter genes in somatic HeLa cells [29], [30], and identifies *Hdac1* as a novel regulator of retrotransposon expression in mouse ES cells. *Hdac1^−/−^* ES cells do not appear to change expression of other classes of repeat probe (Figure 3E), and therefore Hdac1 appears to be more restricted than either DNA methylation or Eset in the range of retrotransposon sequence classes that it affects. However unlike DNA methylation or Eset, Hdac can have both positive and negative effects on expression of retrotransposons. Thus although the Dnmt TKO, *Eset^shRNA^*, and *Hdac1^−/−^* ES cell lines all upregulate individual retrotransposons, these mechanisms appear to have different effects on retrotransposon expression at a genome-wide level.

### Interactions between Retrotransposon Silencing Mechanisms in ES Cells

**Figure 4:**
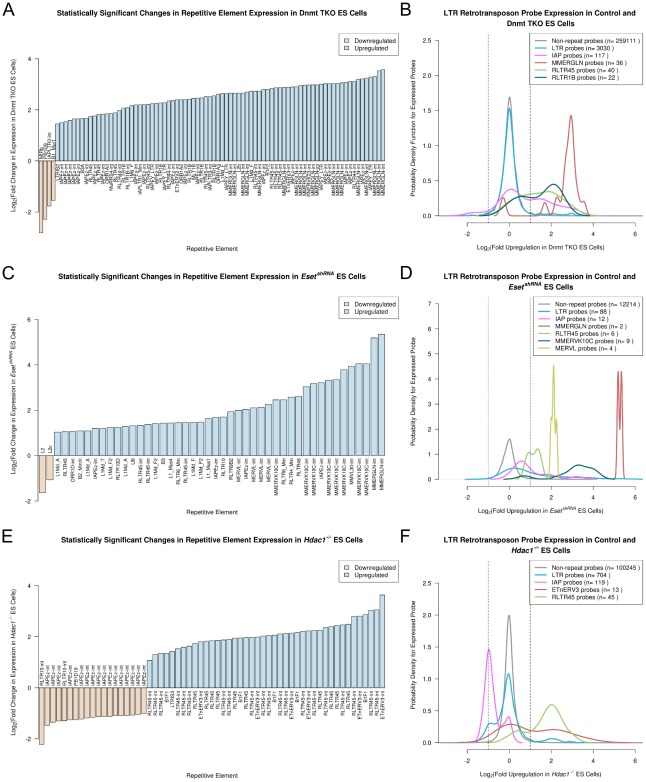
Genome-wide retrotransposon targets of transcriptional repression mechanisms in mouse ES cells. (A, C, E) Histograms showing repeat probes that change expression at least 2 fold (p<0.01) in Dnmt TKO, *Eset^shRNA^*, and *Hdac1^−/−^* ES cells respectively. (B, D, F) Plots showing the behaviour of the selected retrotransposon probe populations in Dnmt TKO, *Eset^shRNA^*, and *Hdac1^−/−^* ES cells respectively. Retrotransposons are colour-coded according to the legend. Vertical lines indicate the 2 fold change cut-off used in panels A, C and E. Note the divergent behaviour of *IAP* and *RLTR45* retrotransposons in *Hdac1^−/−^* ES cells in contrast to Dnmt TKO and *Eset^shRNA^* ES cells.

**Figure 5:**
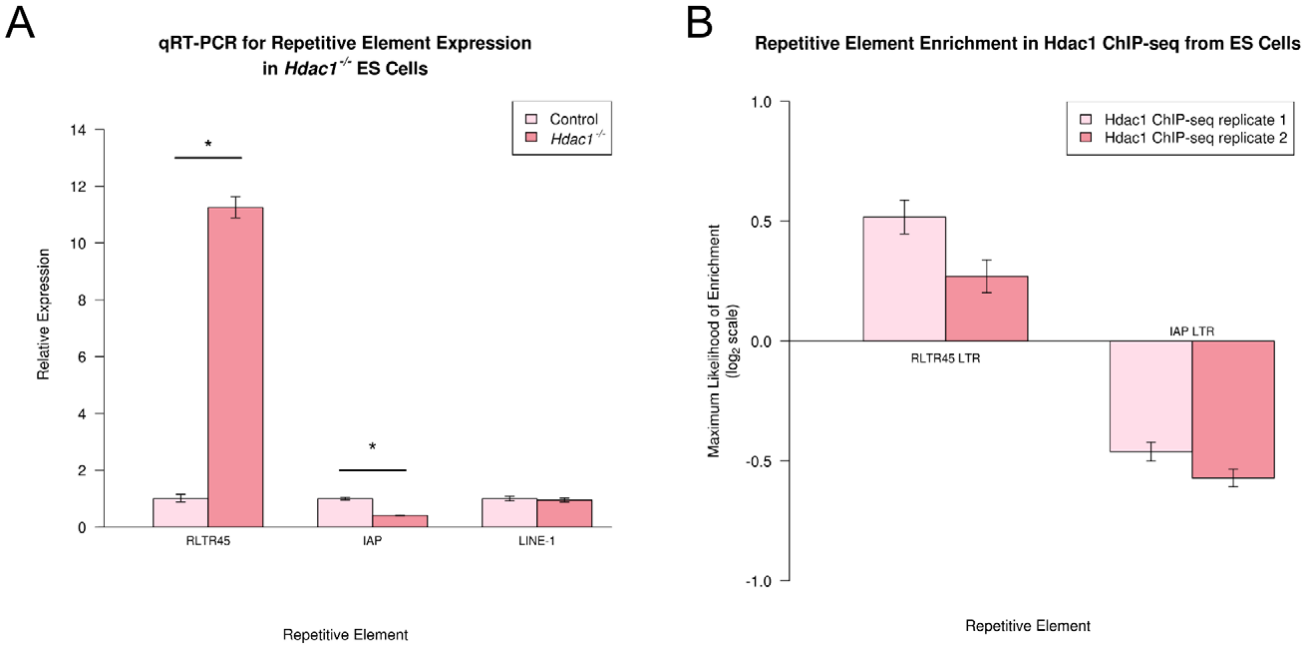
Hdac1 regulates expression of LTR retrotransposons in mouse ES cells. (A) qRT-PCR verification of *LINE-1*, *RLTR45* and *IAP* expression in *Hdac1^−/−^* ES cells. Expression levels (mean ± standard error for three biological replicates) were normalized to *β-Actin* and expressed relative to control ES cells. IAP and LINE1 5′UTR primer sets (Figure S2) were used to assess *IAP* and *LINE-1* expression. Asterisks indicate a statistically significant difference (p<*0.05*) for *RLTR45* and *IAP* elements. *RLTR45* expression is upregulated in *Hdac1^−/−^* ES cells, but IAP expression is downregulated. (B) Enrichment of LTR retrotransposon sequences in Hdac1 ChIP-seq data from mouse ES cells. The maximum likelihood of enrichment (±95% confidence intervals) for *RLTR45* LTR and *IAP* LTR sequences Hdac1 ChIP-seq relative to whole cell extract is shown. *RLTR45* LTR sequences are enriched in the Hdac1 ChIP-seq indicating a physical association between Hdac1 and *RLTR45* retrotransposon chromatin, in contrast *IAP* LTR sequences are depleted.

We next investigated how the Dnmt, Eset and Hdac1 transcriptional repression mechanisms interact in ES cells by identifying distinct and overlapping retrotransposon targets for these mechanisms. We identified repeat probes in each of the Dnmt TKO, *Eset^shRNA^*, and *Hdac1^−/−^* ES cell datasets that changed expression at least 2 fold (p<0.01) relative to the appropriate wild-type control datasets. 84 (0.8%) of the 10,316 expressed repeat probes changed expression at least 2 fold (p<0.01) in the Dnmt TKO ES cells, with multiple probes for *MMERGLN* and *RLTR1B* (ERV1 family), and *IAP* and *RLTR45* (ERVK family) retrotransposons all showing upregulation in these cells (Figure 4A, 4B). These findings correlate well with recent RNA-seq data from Dnmt TKO ES cells: *MMERGLN*, *RLTR1B*, *IAP* and *RLTR45* are all upregulated ∼2.5–13 fold in Dnmt TKO ES cell RNA-seq data [22]. However the two other elements (MMERVK10C and *RMER16*) reported as upregulated >2 fold in Dnmt TKO ES cells by RNA-seq (∼2.3 fold upregulation for each [22]) have no detectable change in expression in the microarray data suggesting that microarray analysis is less sensitive than RNA-seq for detecting some changes in LTR retrotransposon expression. In *Eset^shRNA^* ES cells, 125 (45%) of the 277 expressed repeat probes changed expression at least 2 fold (p<0.01), with multiple probes for *MMERGLN* (ERV1 family), *MMERVK10C, IAP* and *RLTR45* (ERVK family), *MERVL* (ERVL family) and *LINE-1* repetitive elements all showing upregulation in *Eset^shRNA^* ES cells (Figure 4C, 4D). These elements represent a small subset of those reported previously as being upregulated in *Eset^−/−^* ES cells [22], [24], which may reflect greater loss of *Eset* function in *Eset^−/−^* conditional knockout ES cells than in ES cells transiently transfected with knock-down shRNAs. Interestingly, although comparison of the Dnmt TKO and *Eset^shRNA^* ES cell datasets suggests that some retrotransposon sequences (*MMERGLN*, *IAP*, *RLTR45*) are co-repressed by both DNA methyltransferases and Eset histone methyltransferase, analysis of the *Hdac1^−/−^* ES cell data shows striking divergences in the behaviour of these elements (Figure 4E, 4F). 74 (3.7%) of the 1971 expressed repeat probes changed expression at least 2 fold (p<0.01) in *Hdac1^−/−^* ES cells, with multiple probes for the *ETnERV3* and *RLTR45* (ERVK family) retrotransposons showing upregulation in *Hdac1^−/−^* ES cells (Figure 4E, 4F). These elements share considerable sequence similarity at the nucleotide level (84% identity over 4.2 kb of sequence). Interestingly, although *RLTR45* and *IAP* elements both appear to be co-repressed by DNA methyltransferases and Eset histone methyltransferase (Figure 4A–D), multiple probes for *IAP* (ERVK family) retrotransposons behaved quite differently from the *RLTR45* probes and were downregulated in *Hdac1^−/−^* ES cells (Figure 4E, 4F). Although Hdac1 typically acts as a transcriptional repressor, the apparent downregulation of *IAP* elements in *Hdac1^−/−^* ES cells would parallel the behaviour of some single-copy gene targets of Hdac1 [43]. We verified the microarray analysis of LTR retrotransposon expression by performing qRT-PCR on *Hdac1^−/−^* ES cells: significant upregulation of *RLTR45* elements (11 fold, p<0.05) and downregulation of *IAP* elements (2.5 fold, p<0.05) was confirmed using this methodology (Figure 5A). Thus expression of some LTR retrotransposons is perturbed in the absence of *Hdac1* in mouse ES cells. Furthermore, the differences between *RLTR45* and *IAP* expression in *Hdac1^−/−^* ES cells suggests that an *Hdac1*-dependent transcriptional silencing mechanism is being recruited to retrotransposons independently of DNA methyltransferase or Eset histone methyltransferase activity.

The changes in *IAP* and *RLTR45* element expression in *Hdac1^−/−^* ES cells could be an indirect consequence of other gene expression changes that occur in *Hdac1^−/−^* ES cells [43], or may reflect a more direct role for Hdac1 in transcriptional regulation of these elements. To investigate whether RLTR45 and *IAP* are direct targets of Hdac1 in ES cells, we analysed high throughput sequencing data from ES cell chromatin Hdac1 immunoprecipitation (Hdac1 ChIP-seq from mouse ES cells [38]) for enrichment of repetitive element sequences [25]. Interestingly, RLTR45 LTR sequences are enriched in Hdac1 ChIP-seq relative to whole cell extract controls (Figure 5B), suggesting that Hdac1 is negatively regulating *RLTR45* expression in ES cells through physically associating with *RLTR45* LTRs. In contrast *IAP* LTR sequences are depleted in Hdac1 ChIP-seq (Figure 5B), consistent with the downregulation of *IAP* expression in *Hdac1^−/−^* ES cells being an indirect consequence of other changes in gene expression in these cells. Taken together, these data suggest that Hdac1 is directly recruited to *RLTR45* retrotransposons to silence their expression in ES cells.

### Identifying LTR Retrotransposon Targets of Polycomb Repressive Complexes in ES Cells

Our genome-wide analysis of retrotransposon silencing in Dnmt TKO, *Eset^shRNA^*, and *Hdac1^−/−^* ES cells suggests that multiple mechanisms contribute to silencing individual retrotransposon sequences in ES cells. These silencing mechanisms may be recruited sequentially or independently to target sequences. To investigate the interaction between different transcriptional repression complexes at retrotransposon sequences in more detail, we examined retrotransposon silencing in ES cells carrying mutations in components of the polycomb repressive complexes PRC1 and PRC2.

**Figure 6:**
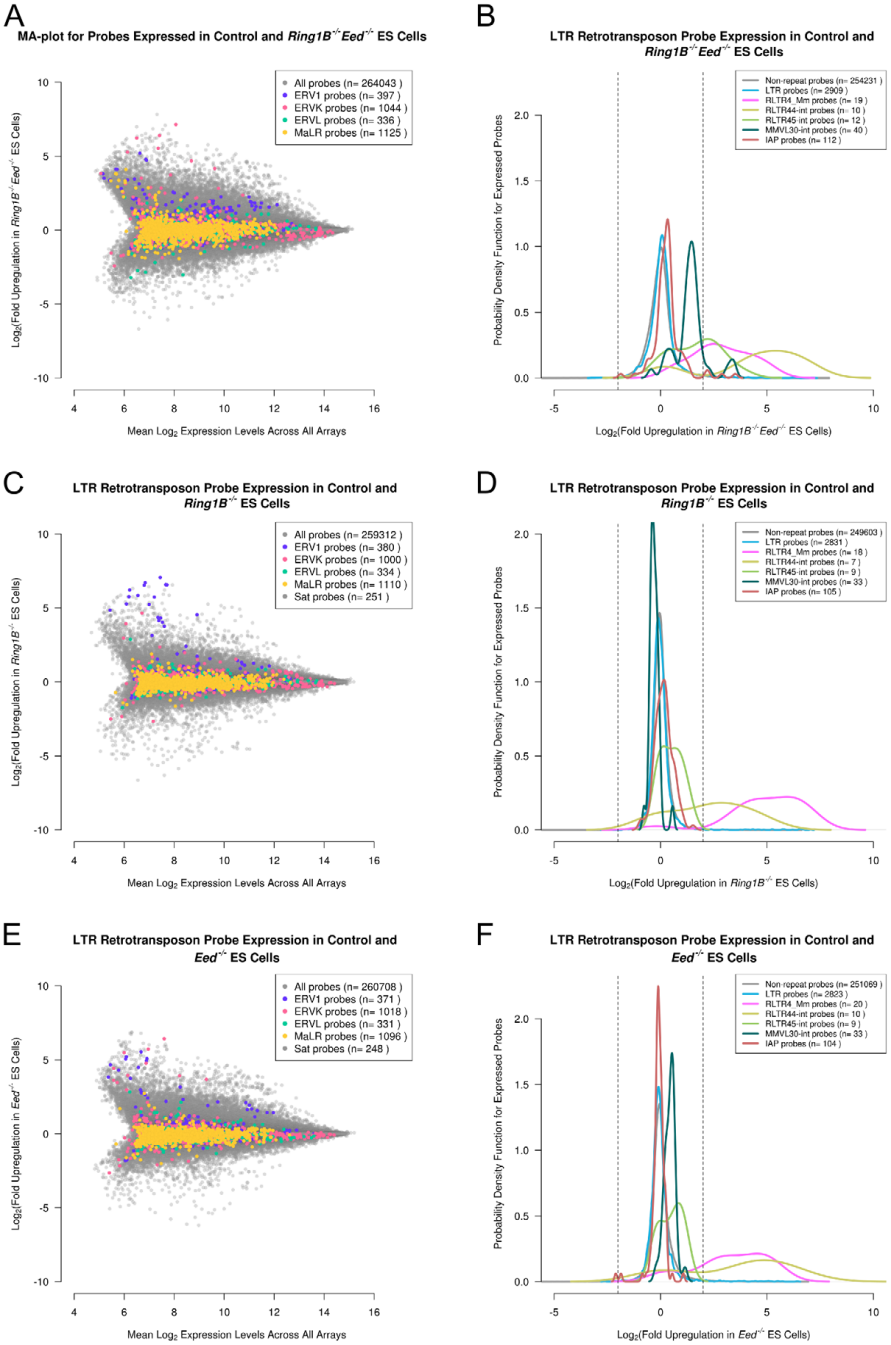
LTR retrotransposon targets of polycomb repressive complexes in ES cells. (A, C, E) MA-plots for *Ring1B^−/−^ Eed^−/−^* double knockout, *Ring1B^−/−^* single knockout and *Eed^−/−^* single knockout *ES* cells showing how different classes of LTR retrotransposons change expression in these cell lines. (B, D, F) Plots showing the behaviour of selected retrotransposon probe populations in *Ring1B^−/−^ Eed^−/−^* double knockout, *Ring1B^−/−^* single knockout and *Eed^−/−^* single knockout *ES* cells. The selected retrotransposons are all represented by multiple upregulated probes (≥4 fold upregulation, p<0.01) in *Ring1B^−/−^ Eed^−/−^* ES cells. Vertical lines indicate a 4 fold change. Note that some retrotransposons (e.g. *MMVL30*, *RLTR45*) are upregulated in double knockout but not single knockout ES cells, other retrotransposons (e.g. *RLTR44*) are upregulated in all three ES cell lines. Retrotransposon probes are colour-coded as shown in the plot legends.

Conventional repression of gene expression by the polycomb repressive complexes PRC1 and PRC2 is thought to involve PRC2 methylating histone H3K27 and sequentially recruiting PRC1 to target loci [reviewed in 46]. However, a recent study on ES cells carrying mutations in the PRC1 component Ring1B, or mutations in the PRC2 component *Eed*, or mutations in both *Ring1B* and *Eed* has suggested that PRC1 and PRC2 are recruited independently and act redundantly to repress *MuLV* and *IAP* repetitive elements in this cell type [26]. We therefore computationally analysed genome-wide retrotransposon silencing in Ring1B*^−/−^*, *Eed^−/−^*, and *Ring1B^−/−^ Eed* ^−/−^ ES cells to determine whether any additional LTR retrotransposons are redundantly regulated by polycomb repressive complexes, and also to test whether any LTR retrotransposons are regulated by conventional sequential targeting of polycomb repressive complexes. *Ring1B^−/−^ Eed^−/−^* ES cells have numerous differences in gene expression compared to wild-type ES cells [26], and although LTR retrotransposon probes do not appear to be preferentially affected by loss of both PRC1 and PRC2 relative to other probes in the dataset, a number of ERV1 and ERVK probes are upregulated in *Ring1B^−/−^ Eed^−/−^* ES cells (Figure 6A). A smaller subset of LTR retrotransposon probes is upregulated in *Ring1B^−/−^* (Figure 6C) and *Eed^−/−^* (Figure 6E) single knockout ES cells. We identified LTR retrotransposon probes that were strongly upregulated at least 4 fold (p<0.01) in *Ring1B^−/−^ Eed^−/−^* ES cells (Figure 6B) and monitored how these LTR retrotransposons behaved in *Ring1B^−/−^* (Figure 6D) and *Eed^−/−^* (Figure 6F) single knockout ES cells. *MMVL30* (ERV1 family) probes were upregulated in *Ring1B^−/−^ Eed^−/−^* double knockout ES cells, but did not change greatly in either *Ring1B^−/−^* or *Eed^−/−^* single knockout ES cells, consistent with these elements being redundantly and independently regulated by PRC1 and PRC2 [26]. A small number of *IAP* probes also appeared to be more strongly upregulated in *Ring1B^−/−^ Eed^−/−^* double knockout ES cells than in either single knockout cell line: 4 of the 112 *IAP* probes that are expressed in this dataset are upregulated at least 4 fold (p<0.01) in *Ring1B^−/−^ Eed^−/−^* double knockout ES cells, but no *IAP* probes are upregulated by these criteria in either single knockout cell line (Figure 6B, 6D, 6F). This is consistent with previous observations that *IAP* elements are redundantly and independently regulated by PRC1 and PRC2 [26]. *RLTR45* (ERVK family) probes are also more strongly upregulated in *Ring1B^−/−^ Eed^−/−^* double knockout ES cells than in either single knockout cell line suggesting that this element is a novel retrotransposon target for redundant silencing by polycomb repressive complexes (Figure 6B, 6D, 6F).

Interestingly, genome-wide analysis of retrotransposon expression also suggests that some LTR retrotransposon probes are being repressed by conventional sequential recruitment of PRC2 and PRC1. *RLTR44* (ERVK family) probes appear to be similarly upregulated in *Ring1B^−/−^ Eed^−/−^* double knockout and single knockout ES cells (Figure 6B, 6D, 6F). The slightly lower upregulation of *RLTR44* probes in *Ring1B^−/−^* ES cells compared to *Eed^−/−^* ES cells may represent Ring1A-containing PRC1 complexes contributing to polycomb-mediated repression in ES cells [47]. RLTR44 retrotransposons do however appear to be a novel retrotransposon target for conventional sequential silencing by polycomb repressive complexes. Thus computational analysis of gene expression in polycomb mutant cell lines suggests that PRC1 and PRC2 interact in different ways on different retrotransposon targets to bring about silencing of these repetitive elements in ES cells.

### Differential Regulation of Retrotransposon Genomic Loci

**Figure 7:**
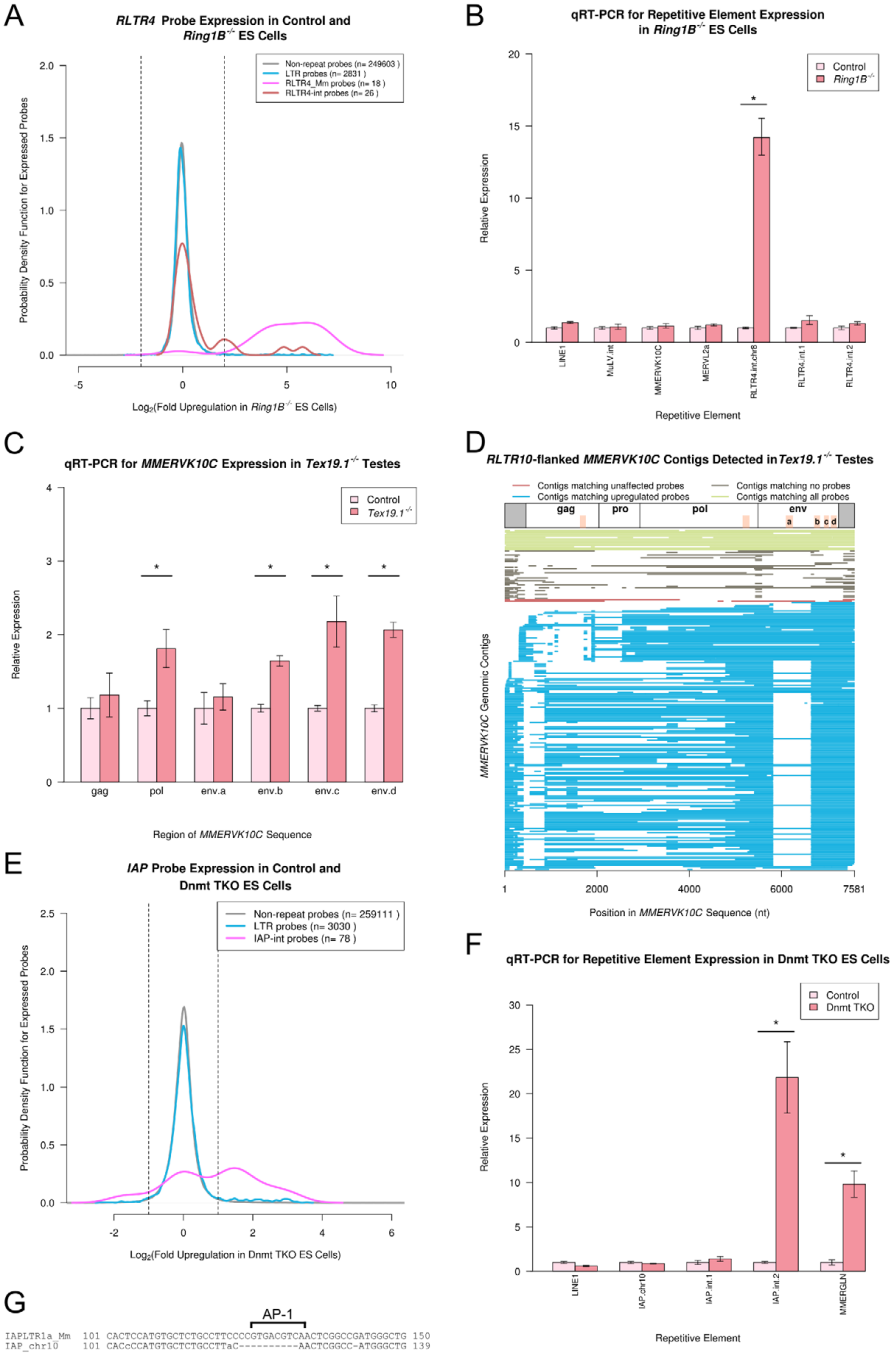
Differential regulation of retrotransposon genomic loci. (A) Plot showing the differential behaviour of different *RLTR4* retrotransposon probe populations in *Ring1B^−/−^* single knockou*t ES* cells. Different *RLTR4* probe populations are colour-coded as shown in the legend, and vertical lines indicate a 4 fold change. (B) qRT-PCR verification of repetitive element expression in *Ring1B^−/−^* ES cells. Expression levels (mean ± standard error) were normalized to *β-Actin* and expressed relative to wild-type control ES cells. MMERVK10C env.c and LINE1 5′UTR primer sets (Figure S2) were used to assess *MMERVK10C* and *LINE-1* expression. The asterisk indicates a statistically significant difference (p<0.05). Note that different primers for *RLTR4* elements behave differently in the qRT-PCR assay. (C) qRT-PCR for different *MMERVK10C* primer sets in *Tex19.1^−/−^* knockout and littermate control testes at 16 dpp. Expression levels (mean ± standard error for three animals) were normalized to *β-Actin* and expressed relative to littermate controls. Asterisks indicate statistically significant differences (p<0.05) (D) Plot showing the *MMERVK10C* genomic contigs flanked by *RLTR10C* LTRs that match only upregulated probes (blue), only unaffected probes (brown), neither class of probes (grey), or both classes of probe (green) in *Tex19.1^−/−^* testes. Each contig is represented by a horizontal line that indicates the regions of the *MMERVK10C* sequence within it. The upregulated *MMERVK10C* contigs appear to contain recurrent deletions and may be non-autonomous. The positions of the qRT-PCR primers used in (C) are shaded orange. (E) Plot showing the bimodal behaviour of *IAP-int* retrotransposon probe populations in Dnmt TKO ES cells. Vertical lines indicate a 4 fold change. (F) qRT-PCR for of repetitive elements in Dnmt TKO ES cells. Expression levels (mean ± standard error) were normalized to *Gapdh* and expressed relative to wild-type control ES cells. The asterisk indicates a statistically significant difference (p<0.05). The LINE1 5′UTR.b primer set (Figure S2) was used to assess *LINE-1* expression. Note the difference in behaviour between the two IAP-int primer sets. The *IAP* contig carrying deletions in the AP-1 binding site shown in panel G (IAP_chr10 primers) is expressed but not upregulated in Dnmt TKO ES cells. (G) Sequence alignment between an LTR of a full-length *IAP* element that does not change expression in Dnmt TKO ES cells (IAP_chr10), and the consensus sequence for the LTR (IAPLTR1a_Mm). The 10 bp deletion removes the AP-1 transcription factor binding site in the LTR.

During analysis of the *Ring1B^−/−^ Eed^−/−^* double knockout and single knockout ES cells, we noticed that probes for *RLTR4* retrotransposons were strongly upregulated in all three cell lines (Figure 6B, 6D, 6F). However the *RLTR4* probes that are upregulated correspond mainly to the LTR region (*RLTR4_Mm*) but usually not the internal region (*RLTR4-int*) of this element (Figure 7A). This suggests that the upregulation of these probes may represent expression from a subset of *RLTR4* loci, possibly corresponding to truncated or chimaeric elements. We therefore mapped the genomic location of the *RLTR4* LTR and internal probes that were upregulated in *Ring1B^−/−^* ES cells back onto the genome using BLAT. In contrast to the retrotransposon probes upregulated in other datasets analysed in this study, the *RLTR4* probes upregulated in *Ring1B^−/−^* ES cells did not map to multiple genomic loci. Rather, all of the upregulated *RLTR4* probes mapped only to a single *RLTR4*-containing genomic locus on chromosome 8 (chr8:125949704–125958431). The *RLTR4* probes that did not change expression in *Ring1B^−/−^* ES cells mapped to multiple loci in the genome. Thus the upregulation of a subset of *RLTR4* probes in *Ring1B^−/−^* ES cells may represent upregulation of a single genomic copy of this element. This locus appears to contain *RLTR4-int* and *MuLV-int* sequences flanked by *RLTR4_Mm* sequences that each contains an inversion and a ∼200 bp deletion relative to the 742 bp consensus sequence. qRT-PCR using primers designed to specifically detect the *RLTR4-int* sequence at this locus confirmed that expression of this region is strongly upregulated in *Ring1B^−/−^* ES cells (Figure 7B), whereas qRT-PCR using primer sets that recognize multiple copies of *RLTR4-int* suggest that these elements are, in general, not upregulated in *Ring1B^−/−^* ES cells (Figure 7B). qRT-PCR also confirmed that representative ERV1, ERVK and ERVL LTR retrotransposons were not changing expression in *Ring1B^−/−^* ES cells (Figure 7B), consistent with the computational analysis. The divergent copy of *RLTR4* on chromosome 8 appears to be silenced by conventional polycomb repression as it is de-repressed in both *Ring1B^−/−^* and *Eed^−/−^* single knockout ES cells (Figure 6). This copy of *RLTR4* could have acquired *Ring1B* target sequences through mutations and re-arrangement to make it a target for conventional polycomb silencing. However as *RLTR4* is derived from *MuLV* [48], a target of redundant silencing by PRC1 and PRC2 [26], it is perhaps more likely that changes in this divergent copy of *RLTR4* have removed sequences that allow PRC2-independent silencing of this locus by PRC1, making it behave as a conventional target for polycomb repression.

Many of the changes in retrotransposon expression that we have characterized in ES cells and germ cells involve subsets of probes for particular retrotransposons changing expression (Figure 1, Figure 4, Figure 6) suggesting that different genomic copies of these retrotransposons may be differentially regulated in these cell types. In *Tex19.1^−/−^* testes, six of the nine expressed *MMERVK10C* probes in the dataset are upregulated at least 2 fold (Figure 1). All six of the upregulated *MMERVK10C* probes are located in the *MMERVK10C env* open reading frame. Two of the remaining three *MMERVK10C* probes are also located in the *env* gene and are upregulated in *Tex19.1^−/−^* testes, but are just below the 2 fold change threshold. The single *MMERVK10C* probe that is located in the *gag* region does not significantly change expression in the *Tex19.1^−/−^* testis dataset. We validated the computational data by qRT-PCR and confirmed that the *gag* and *env* regions of *MMERVK10C* are indeed differentially sensitive to loss of *Tex19.1* in mouse testes (Figure 7C). Interestingly, we noted that primer sets designed to different parts of *MMERVK10C env* (env.a – env.d) were also differentially sensitive to loss of *Tex19.1* (Figure 7C). These data suggest that a subset of *MMERVK10C* loci may be upregulated in *Tex19.1^−/−^* testes. Cloning and sequencing multiple independent clones of the env.c PCR product confirmed that multiple *MMERVK10C* loci were expressed in *Tex19.1^−/−^* and control testes (data not shown). The *pol* sequence is not covered by probes on the array but this region of *MMERVK10C* is also significantly upregulated in *Tex19.1^−/−^* testes (Figure 7C). Although *in silico* PCR suggests that the different *MMERVK10C* primer sets detect different numbers of *MMERVK10C* loci (*gag* primers detect 95 loci, *pol* primers detect 164 loci, *env.a* – *env.d* primers detect 78,70,179 and 40 loci respectively), the qRT-PCR data suggest that expression of these amplicons is differentially affected by loss of *Tex19.1*. We investigated the differential regulation of *MMERVK10C gag* and *env* regions by mapping the six strongly upregulated *env* probes and the single unaffected *gag* probe to individual *MMERVK10C* genomic loci, and assembled the *MMERVK10C* genomic loci into contigs. As *MMERVK10C* sequences that have retained flanking *RLTR10* LTRs are more likely to be transcriptionally active we selected *RLTR10*-flanked *MMERVK10C* contigs for further analysis (Figure 7D). Only 18 of the 250 *RLTR10*-flanked *MMERVK10C* contigs (7%) that we identified in the mouse genome are approximately full-length (contain >95% of *MMERVK10C* reference sequence). Interestingly, many of the *RLTR10*-flanked *MMERVK10C* contigs contain recurrent deletions: one recurrent deletion in the upregulated *MMERVK10C* contigs removes the start of the *gag* open reading frame (nucleotides 399–870 deleted in 33% of these contigs) and appears to be associated with recurrent deletions in *env* (nucleotides 5810–6646 deleted in 33% of all contigs, 5810–6651 deleted in 20% of contigs). The presence of recurrent deletions in the *MMERVK10C* open reading frames at distinct genomic loci suggests that transcripts carrying these deletions may be actively retrotransposing, presumably in a non-autonomous manner through the activity of endogenous retroviral proteins provided *in trans*. The upregulated probes appeared to be highly representative of the *RLTR10*-flanked *MMERVK10C* loci, with 197 of the 250 *RLTR10*-flanked *MMERVK10C* contigs matching only the upregulated probes (Figure 7D). No *RLTR10*-flanked *MMERVK10C* contig matched all upregulated probes, or all the upregulated qRT-PCR primer sets, suggesting that multiple genomic copies of *MMERVK10C* are upregulated in *Tex19.1^−/−^* testes. In contrast, only two *RLTR10*-flanked *MMERVK10C* contigs matched only the unaffected probe (Figure 7D). Interestingly, 12 of the 15 *RLTR10*-flanked *MMERVK10C* contigs that matched both sets of probes were approximately full-length sequences, whereas the contigs that matched only the upregulated probes usually contained deletions with recurrent breakpoints. (Figure 7D). Furthermore, qRT-PCR primers designed to amplify sequences within the 5810–6646 deletion (env.a) do not change expression in *Tex19.1^−/−^* testes, but those amplifying *env* sequences outside this deletion (env.b, env.c, and env.d) are upregulated (Figure 7C, 7D). Thus de-repression of specific subsets of *MMERVK10C* loci could be contributing to the differential regulation of different regions of *MMERVK10C gag* and *env* amplicons in *Tex19.1^−/−^* testes (Figure 7C). The upregulated pol and env.b/env.c primer sets can detect expression from *RLTR10*-flanked *MMERVK10C* contigs encoding intact pol and env proteins respectively (>90% of open reading frame intact relative to *MMERVK10C* reference sequence), but not contigs where the gag, pol, pro and env proteins are all intact. This suggests that the upregulated *MMERVK10C* transcripts may have some protein coding potential, but may need to rely on proteins provided *in trans* for retrotransposition. Some of the deletions in the upregulated *MMERVK10C* contigs, particularly the consistent disruption to parts of the *gag* region, may be removing sequences used to recruit *Tex19.1*-independent retrotransposon silencing mechanisms. These loci would therefore be more reliant on the *Tex19.1-*dependent pathway for repression in germ cells, and be specifically de-repressed in *Tex19.1^−/−^* testes. Thus the differential regulation of *MMERVK10C* probes in *Tex19.1^−/−^* testes may be caused by the emergence of variant non-autonomous *MMERVK10C* elements that have deleted the sequences used to target silencing mechanisms to *MMERVK10C*.

We noted that *IAP* retrotransposon probes in Dnmt TKO ES cells lines were also exhibiting bimodal behaviour (Figure 4B). To investigate whether this represents differential regulation of *IAP* loci we designed qRT-PCR primers to *IAP* loci matching either upregulated or unaffected *IAP-int* probes (Figure 7E). qRT-PCR confirmed that some *IAP* loci are upregulated in Dnmt TKO ES cells, whereas others do not change expression (Figure 7F). As expected from the computational analysis of retrotransposon expression in Dnmt TKO ES cells, expression of *LINE-1* elements do not change in Dnmt TKO ES cells, and *MMERGLN* elements are upregulated, when assessed experimentally by qRT-PCR (Figure 7F). Our finding that different genomic copies of *IAP* may be differentially sensitive to loss of DNA methyltransferases is consistent with recent findings from RNA-seq of Dnmt TKO ES cells [22]. A simple interpretation of this phenomenon would be that the IAP loci that are not changing expression in Dnmt TKO ES cells are divergent defective copies of the *IAP* element. However, the unaffected *IAP-int* probes are detecting some *IAP* expression in ES cells, albeit at a lower level than the upregulated probes, suggesting that the *IAP* loci that are detected by the unaffected *IAP-int* probes are not all transcriptionally inert. To investigate why some *IAP* loci are insensitive to DNA methylation we identified the genomic *IAPEz-int* contigs that matched either the upregulated or the unaffected *IAP-int* probes. Although many of the contigs that only matched the unaffected *IAP-int* probes carried large deletions, one locus (chr10:22250294–22243066) contained a relatively intact *IAPEz-int* region flanked by *IAP* LTRs. Interestingly both of the LTRs at this locus contain a small 10 bp deletion (Figure 7G) that removes the conserved AP-1 transcription factor binding site [49]. Only 5 of the 16141 *IAP* LTRs in the mouse genome carry this, or a similar, deletion of the AP-1 binding site, and none of the *IAP* contigs that only match upregulated *IAP-int* probes contain this deletion in their LTRs. We confirmed that this copy of *IAP* (IAP_chr10) was not upregulated in Dnmt TKO ES cells by qRT-PCR (Figure 7F). However, mRNA from this locus was readily detected in wild-type and Dnmt TKO ES cells, suggesting that this copy of *IAP* is constitutively expressed. Loss of the AP-1 binding site in the *IAP* LTRs at this locus therefore does not appear to silence expression of this element, but may render this locus insensitive to regulation by DNA methylation. Interestingly, DNA methylation has been shown to inhibit binding of AP-1 to gene promoters [50]. Inhibition of AP-1 binding to IAP LTRs may therefore be contributing to DNA methylation-mediated repression of *IAP* elements in mouse ES cells.

Taken together, computational analysis of genome-wide retrotransposon silencing suggests that individual loci for a particular retrotransposon can have different sensitivities to retrotransposon suppression mechanisms. Mapping the changes that are present in differentially regulated loci may help to identify *cis*-acting retrotransposon sequences that are being used to recruit silencing mechanisms.

## Discussion

### Evaluation of the Microarray Repeat-Annotation Approach

In this manuscript we describe a simple computational approach to monitor repetitive element expression in microarray gene expression data. We have used repeat-annotation of pre-existing datasets to identify retrotransposons regulated by DNA methylation and different histone modifications in mouse ES cells (Table 3). We have verified that repeat probes present in gene expression microarrays are accurately reporting repetitive element expression by confirming our findings from *Tex19.1*, *Ring1B* and Dnmt TKO microarray analyses by qRT-PCR. In general there appears to be good qualitative correlation between repeats that we identified as changing expression in microarray datasets, and our qRT-PCR verification. Importantly there is also good correlation between repeat probes that are not changing expression in the microarray datasets and our qRT-PCR verification of these repetitive elements. Furthermore, we have used this approach to identify Hdac1 as a component of the retrotransposon silencing machinery in mouse ES cells (Figure 3, Figure 4). Application of this methodology to gene expression microarray data is likely to generate new insights into retrotransposon regulation in mammals, and help to identify further components of the defence mechanisms that protect the mammalian genome from retrotransposition. Consistent with previous re-annotation workflows designed to remove non-informative probes from microarray analyses [40], we found that commercially available mouse gene expression microarray platforms contain a number of probes that map to repetitive regions of the genome. Although expression information from these probes can be discarded to improve analysis of gene expression in the remaining dataset [40], we show here that the information from these probes can be extracted to accurately monitor repetitive element expression.

**Table 3:** Summary of changes in repetitive element expression detected by microarray repeat-annotation in this study.

		ES cells					Testes
		Dnmt TKO	*Eset^shRNA^*	*Hdac1* ^−*/*−^	*Ring1B* ^−*/*−^	*Ring1B^−/−^ Eed^−/−^*	*Tex19.1^−/−^*
**ERV1**	*MMERGLN*	↑	↑	-	-	-	-
	*RLTR1B*	↑	-	-	-	-	-
	*RLTR4*	-	-	-	(↑)	(↑)	-
	*MMVL30*	-	-	-	-	↑	-
**ERVK**	*IAP*	↑	↑	↓	-	(↑)	-
	*RLTR44*	-	-	-	↑	↑	-
	*RLTR45*	↑	↑	↑	-	↑	-
	*MMERVK10C*	-*	↑	-	-	-	↑
	*ETnERV3*	-	-	↑	-	-	-
**ERVL**	*MMERVL*	-	↑	-	-	-	-
**LINE**	*LINE-1*	-	↑	-	-	-	-

Statistically significant upregulation and downregulation of repetitive element expression in mutant ES cell lines or testes is indicated by up and down arrows respectively. Changes that only appear to affect a small number of probes for a repetitive element are indicated in brackets. The degree of change in gene expression detected for these elements is detailed in the main text.

*: Although changes in *MMERVK10C* expression were not detected in Dnmt TKO ES cell microrray data in this study, RNA-seq analysis suggests that some genomic copies of *MMERVK10C* are upregulated in Dnmt TKO ES cells [22].

Repeat-annotation of microarray data can significantly expand the repertoire of repetitive elements studied in an experiment compared to testing selected representative candidates. Indeed this study has identified new target retrotransposons for polycomb repressive complexes and Hdac1 histone deacetylase in mouse ES cells. Although the range of repetitive elements analysed by microarray repeat-annotation will not be as wide as that analysed by RNA-seq [22], [23], between one and two thirds of all retrotransposons in the mouse genome are represented by probes on the microarray platforms that we have analysed here. A direct comparison between microarray repeat annotation (this study) and RNA-seq [22] for detecting changes in retrotransposon expression in Dnmt TKO ES cells shows good correlation between these methods (the four retrotransposons detected as upregulated by microarray analysis are the four most strongly upregulated retrotransposons detected by RNA-seq). However, two additional LTR retrotransposons were detected as upregulated in Dnmt TKO ES cells only by RNA-seq, despite representation of these elements on the microarray. Thus microarray analysis may be less sensitive than RNAseq for detecting some changes in LTR retrotransposon expression, particularly when only a small number of genomic copies are changing expression [22]. In addition, although we have focused on retrotransposon silencing in mouse germ cells and pluripotent cells, the computational approach that we describe here can be readily applied to microarray data from human cells and tissues to inform on retrotransposon expression in relation to retrotransposition in somatic mosaicism [12], [37], epigenetic changes in cancer [51], [52], reprogramming somatic cells into iPS cells [53], and toxicological insults [54]. As repeat-annotation can be applied to pre-existing microarray data as well as new datasets, this methodology can be used to extract information from many of the ∼18,000 microarray gene expression data series that have been generated and deposited in publicly available databases [38]. This makes repeat-annotation of microarray data an attractive approach to test hypotheses and generate initial findings upon which more detailed research can be built. Thus microarray repeat-annotation represents a simple and cost-effective addition to the methods available to study repetitive element silencing at a genome-wide level.

### Differential Regulation of Specific Genomic Copies of a Retrotransposon

One of the features of the computational approach that we have outlined here is that our analysis is based on aligning probe sequences to Repeatmasked regions of the genome, rather than to Repeatmasker consensus sequences. If different genomic copies of a repetitive element are behaving in different ways in an experiment then repeat-annotation of microarray data can potentially monitor expression from divergent genomic copies of a repetitive element. Clearly the extent to which multiple genomic copies of a particular element can be monitored will depend on the coverage of probes for that element. In the Affymetrix Mouse Expression 430 2.0 GeneChip platform that contains ∼26,000 repeat probes we have been able to detect differential regulation of different genomic copies of *RLTR4* elements in *Ring1B^−/−^* ES cells, *IAP*, *RLTR45* and *RLTR1B* elements in Dnmt TKO ES cells and *ETnERV3* and *RLTR45* in *Hdac1^−/−^* ES cells. Remarkably, for *Ring1B^−/−^* ES cells we were able to detect expression changes that are possibly arising from only a single divergent copy of *RLTR4*. Thus repeat-annotation of microarray data appears to be able to monitor expression from divergent genomic copies of a repetitive element.

For *MMERVK10C* elements, analysis of the genomic loci matching retrotransposon probes was able to generate some insight into why some genomic copies of these elements are more sensitive to loss of suppression mechanisms than others. Loss of parts of the *gag* or *env* regions of *MMERVK10C* may be associated with genomic copies becoming more sensitive to *Tex19.1*-dependent suppression in male germ cells (Figure 7D). Interestingly, non-autonomous variants of *IAP* (*IAPΔ1*) that carry deletions in the *gag* region retrotranspose more frequently than their full-length counterparts [55]. Thus sequences in the *gag* region of both *IAP* and *MMERVK10* may be being used by host defence mechanisms to target these elements for silencing. In addition, analysis of differentially regulated *IAP* loci allowed us to identify a region in the *IAP* LTR that may be targeted by host silencing mechanisms (Figure 7G). DNA methylation at this conserved AP-1 transcription factor binding site may contribute to Dnmt-dependent repression of *IAP* elements in ES cells by inhibiting AP-1 binding. However, further experimental work is needed to functionally characterize the consequences of these deletions for *MMERVK10C* and *IAP* silencing in germ cells and ES cells. Our analysis of *MMERVK10C* and *IAP* elements suggests that the behaviour of sequence variants in a retrotransposon's population can potentially be used to identify *cis*-acting sequences involved in retrotransposon suppression. In this respect, although repeat-annotation of microarray data may give some indication of differential regulation of repeat loci, RNA-seq may potentially be a more powerful approach to identify which genomic copies of an element are responsible for changes in expression.

As with all studies reporting changes in retrotransposon expression, determining whether changes in RNA or protein levels are caused by misregulation of one copy or many copies of a retrotransposon can be difficult. However, determining the sequence of the retrotransposon loci or transcripts that change expression in microarray datasets is an important prerequisite for assessing the functional potential of the mis-expressed retrotransposons. Finer sub-classification of repeat probes to distinguish between expression of functional and non-functional copies of a retrotransposon, for example active and inactive *LINE-1* elements, may not be accurate due to the short length of microarray probes: longer sequences are usually required to unambiguously identify a particular retrotransposon subfamily. Furthermore, none of the *LINE-1* probes present in the Illumina and Affymetrix arrays analysed here match the consensus monomer sequences that distinguish active Tf, Gf and A-type *LINE-1* elements. Thus microarray repeat-annotation may not be able to distinguish whether functional or non-functional genomic copies of a particular retrotransposon are deregulated, but may be useful in identifying subpopulations of genomic copies that include or exclude the misregulated retrotransposon sequence.

### Regulation of Retrotransposon Expression in Mouse ES Cells and Germ Cells

We have used repeat-annotation of microarray data to investigate whether some of the established mechanisms for retrotransposon silencing have additional retrotransposon targets in mouse ES cells. This analysis has demonstrated that there is a complex interplay between DNA methylation and histone modifications regulating the expression of the spectrum of repetitive elements in the mouse genome (Table 3). The LTR retrotransposons that we identified as being upregulated in Dnmt TKO ES cells overlap well with those identified recently by RNA-seq of Dnmt TKO ES cells [22]. Interestingly, many repetitive elements that belong to the same ERVK LTR retrotransposon family as IAP elements were not upregulated in Dnmt TKO ES cells suggesting that related retrotransposons can differ in their sensitivity to DNA methylation. Similarly, our finding that *MMERVK10C*, but not closely related retrotransposons such as *IAP*, are upregulated in *Tex19.1^−/−^* testes suggests that closely related retrotransposons differ in sensitivity to regulatory mechanisms in developing germ cells as well as ES cells. The differential behaviour of *IAP* and *MMERVK10C* elements in *Tex19.1^−/−^* testes could be caused by differences in the availability of transcriptional factors or by differences in silencing mechanisms associated with these elements. However as *IAP* LTRs are able to drive expression in spermatogonia [8], [19], which are present in the 16 dpp Tex19.1*^−/−^* testes analysed here, the differential behaviour of *IAP* and *MMERVK10C* in *Tex19.1^−/−^* testes may reflect differences in silencing mechanisms acting on these elements. DNA methylation plays an important role in silencing *IAP* elements in spermatogonia [19], and redundancy between silencing mechanisms may well be contributing to the differential behaviour of MMERVK10C and *IAP* elements in *Tex19.1^−/−^* testes. Some of the retrotransposon targets for DNA methylation, *Tex19.1*, and the other silencing mechanisms that we have studied, may be obscured by redundancy between silencing mechanisms, and each of the mechanisms that we have studied here may have a broader range of targets than we have been able to identify.

Like *IAP* elements, the *RLTR45* ERVK LTR retrotransposon and the *MMERGLN* and *RLTR1B* ERV1 LTR retrotransposons are all upregulated in Dnmt TKO ES cells. The level of upregulation of *IAP*, *MMERGLN*, *RLTR45* and *RLTR1B* retrotransposons in Dnmt TKO ES cells was relatively low, consistent with previous observations for *IAP* elements [21]. Additional mechanisms are likely to play a role in transcriptionally repressing these retrotransposons in ES cells, and Kap1/Eset-mediated repression appears to be one of the silencing pathways that plays a prominent role in repression of these elements [this study], [22,23]. At least for *IAP* elements, differentiated cells may rely more heavily on DNA methylation than Kap1/Eset for repression [21], [23]. It will be interesting to test whether transcription of *MMERGLN*, *RLTR1B* and *RLTR45* repetitive elements is directly regulated by DNA methylation, and whether DNA methylation plays a dominant role in repressing these repetitive elements in differentiated cells.

*MMERGLN* and *RLTR45* elements behaved similarly to *IAP* elements in *Eset^shRNA^* ES cells. Our finding that *MMERGLN*, *RLTR45* and *MMERVK10C* are all upregulated in *Eset^shRNA^* ES cells is consistent with these elements being enriched for H3K9Me3 in ES cells [22], [25], and with recent RNA-seq and ChIP-seq data from *Eset^−/−^* ES cells [22]. We also found that *MERVL-int* elements were upregulated in *Eset^shRNA^* ES cells. These elements have also been reported to be upregulated in *Kap1^−/−^* ES cells [23]. ERVL retrotransposons are enriched for H3K27Me3 but not H3K9Me3 in ES cells [25], and the upregulation of *MERVL-int* (this study) and *MTA* [24] ERVL retrotransposons in Eset*^shRNA^* and *Eset^−/−^* ES cells may be an indirect effect of loss of *Eset* function. As ES cells lacking *Eset* differentiate towards the trophectoderm lineage [42], some of the changes in gene expression in *Eset^shRNA^* and *Eset^−/−^* ES cells may be an indirect consequence of this change in cell fate, or indeed any other change in gene expression. Indeed all of the microarray analyses of gene expression in ES cells that we have repeat-annotated are subject to the caveat that some changes in gene expression in these datasets may be consequences of differences in the proportion or type of differentiated cells present in the ES cell cultures. Further experiments will be required to determine why some ERVL retrotransposons are modestly upregulated in *Eset^shRNA^* and *Eset^−/−^* ES cells.

Importantly this study also identifies the histone deacetylase Hdac1 as a regulator of retrotransposon expression in mouse ES cells. The HDAC family of histone deacetylases has been implicated in retrotransposon suppression in some cell types [28], [44], [45], and HDAC1 has been shown to suppress expression from avian retroviral LTRs in somatic HeLa cells [29], [30]. The microarray analysis that we present here extends these findings by identifying the retrotransposon elements that are regulated by Hdac1 in mouse ES cells. Interestingly, although *RLTR45* and *IAP* elements behaved similarly in Dnmt TKO and *Eset^shRNA^* ES cells, these elements were misregulated in opposite directions in *Hdac1^−/−^* ES cells. Thus the silencing mechanisms operating on repetitive elements appear to be modular, with different combinations of mechanisms acting on different elements (Table 3). Furthermore, these data suggest that the *Hdac1*-mediated and Dnmt-mediated silencing mechanisms operating on these elements are being targeted independently to *RLTR45* and *IAP* retrotransposons. The upregulation of *RLTR45* elements in *Hdac1^−/−^* ES cells, together with the enrichment of *RLTR45* sequences in Hdac1 ChIP-seq data from ES cells, suggests that an Hdac1-containing repressor complex may be recruited to *RLTR45* loci and silence this element. Further analysis of Hdac1-binding and histone modification at *RLTR45* elements is likely to generate more mechanistic insight into this silencing event. The downregulation of *IAP* elements in *Hdac1^−/−^* ES cells parallels the behaviour of some endogenous genes in these ES cells [43]. It will be informative to determine whether Hdac1 is acting directly on *IAP* elements to promote their transcriptional activation, or the increased activity of Hdac2 in *Hdac1^−/−^* ES cells is responsible for downregulation of *IAP* elements [43]. Interestingly, *LINE-1* elements did not appear to be upregulated in *Hdac1^−/−^* ES cells (Figure 5A), which contrasts with Hdac1's role in repressing *LINE-1* elements in neural stem cells [56]. Again, further experiments will be required to distinguish whether this difference reflects different chromatin environments between pluripotent ES cells and somatic neural stem cells, an effect of different Sox2-interacting partners in these cell types, or redundancy between multiple pathways operating to suppress *LINE-1* activity in ES cells.

In summary we have shown that genome-wide silencing of repetitive elements can be monitored by extracting this information from microarray gene expression data, revealing a complex interplay between mechanisms that act to control retrotransposon expression in mouse ES cells and germ cells, and important differences in the behaviour of different genomic copies of individual retrotransposons. This computational approach has expanded our knowledge of retrotransposon targets for known silencing mechanisms, identified Hdac1 as a regulator of retrotransposon expression in ES cells, and demonstrated that epigenetic silencing mechanisms are independently recruited to retrotransposons in a modular manner.

## Materials and Methods

### Ethics Statement

Animal work was conducted according to UK Home Office regulations and local guidelines for animal welfare.

### Animals

Mice were housed and bred according to UK Home Office regulations and local guidelines for animal welfare. *Tex19.1^−/−^* mice [39] were backcrossed three times to inbred C57BL/6 mice to reduce genetic variation prior to microarray analysis. Animals were culled at 16 days post partum (dpp) by cervical dislocation and testes from Tex19.1*^−/−^* experimental mice and *Tex19.1* ^+/+^ and *Tex19.1* ^+/−^ control littermates were frozen on liquid nitrogen prior to RNA isolation.

### ES Cell Culture

*Ring1B^−/−^* feeder-dependent ES cells [57] were cultured at 37°C, 5% CO2 on a mitomycin C-treated mouse embryonic fibroblast feeder layer, feeder-independent Dnmt TKO and *Hdac1^−/−^* ES cells [20], [43] were cultured at 37°C, 5% CO2 on gelatinized tissue-culture flasks. *Ring1B^−/−^* and *Hdac1^−/−^* ES cells were cultured using DMEM (Invitrogen) supplemented with 15% fetal calf serum, 1× non-essential amino acids, 1 mM sodium pyruvate, 100 units/mL penicillin, 0.1 mg/mL streptomycin, 50 µM β-mercaptoethanol and leukemia inhibitory factor. Dnmt TKO ES cells were cultured using GMEM (Invitrogen) with 10% fetal calf serum rather than DMEM with 15% fetal calf serum. ES cells were harvested using trypsin-EDTA, then pelleted for RNA isolation.

### RNA Isolation and Illumina Beadarray Gene Expression Profiling

RNA was isolated from 16 dpp testes or ES cell pellets using TRIzol (Invitrogen) according to the manufacturer's instructions. RNA was treated with DNAseI (Roche) for 2 h at 37°C to remove genomic DNA contamination. For Illumina Beadarrays of 16 dpp testes, cRNA samples were prepared using Illumina TotalPrep RNA Amplification Kit (Ambion) and hybridized to Illumina Mouse WG-6 v2.0 Beadarrays according to the manufacturers' protocols. The raw and processed *Tex19.1* microarray data have been deposited in the publicly accessible GEO database [38], accesssion number GSE30461.

### qRT-PCR

cDNA synthesis was performed on DNaseI-treated RNA using random primers and Superscript III reverse transcriptase (Invitrogen) according to the manufacturer's instructions. qRT-PCR was performed using Brilliant II/III SYBR Green QPCR Master Mix (Agilent Technologies) or Quantitect SYBR Green detection kit (Qiagen) and a CFX96 Real-Time PCR Detection System (Bio-Rad). Relative changes in gene expression were calculated by normalizing gene expression levels from different samples to *β-Actin* or *Gapdh* as indicated. Expression levels in experimental samples are expressed relative to wild type ES cells or littermate controls. Three technical replicates were performed for each biological sample, and cDNA prepared from each RNA sample in the absence of reverse transcriptase showed no significant qRT-PCR signals. For qRT-PCR of 16 dpp testes, the Sertoli cell-expressed *Sdmg1* gene [58] was used to verify normalization between animals. A two-tailed t-test was used to determine statistical significance of qRT-PCR gene expression changes. The sequences of the primers used for qRT-PCR are available with the online version of this paper (Figure S2).

### Repeat Annotation of Illumina Probes

The DNA sequences of the 50-mer probes used in Illumina Mouse WG-6 Beadchips were downloaded from the manufacturer's website [59]. For each Beadchip version the probe sequences were used to search the mm9 release of the mouse genome by individual chromosome using BLAT [60]. The BLAT parameters used were -minIdentity = 95 -stepSize = 5 -repMatch = 2253. Experimental data suggests that the 50 nt Illumina Beadchip probes will hybridize to mRNAs containing 2 mismatches in the probe sequence with an efficiency of greater than 90% [61]. A *Perl* script was used to compare the genome co-ordinates of the top hit (with a 48/50 nt identity minimum cut-off) for each probe sequence with the co-ordinates of the Repeatmasked regions of the mm9 release of the mouse genome downloaded from the UCSC genome browser [62]. Probes that overlapped a Repeatmasked region by at least 48 nt, and were in the appropriate orientation to recognize sense transcripts were selected and annotated with the Repeatmasker class, family and element corresponding to that genomic region. Tables containing the repetitive element probes for each Illumina Beadchip were imported into R [63] and used to annotate Illumina Beadchip gene expression data. These annotation tables are available with the online version of this paper (Datasets S1, S2, S3).

### Pre-processing of Illumina Beadchip Gene Expression Data

Illumina Beadchip gene expression data for *Eset* shRNA knock-down ES cells were downloaded from the NCBI GEO repository [38], accession number GSE17439 [42]. All analysis of Illumina Mouse Whole Genome WG-6 Beadchip microarrays was performed on probe-level data. Probe-level expression data were background-subtracted in Illumina Beadstudio, then imported into the *lumi* Bioconductor package [64] in R. The data were then log-transformed, and quantile-normalized in *lumi*. The expression data and present/absent calls were exported from *lumi* and any probes that were called as absent in all samples in the experiment were removed from the dataset.

### Repeat Annotation of Affymetrix Probes

These Affymetrix Murine Genome U74Av2 and Mouse Expression 430 2.0 GeneChips contain ∼12,000 and ∼45,000 probesets respectively, with each probeset containing ∼11 different 25 nt probes targeting a specific transcript. The DNA sequences of the 25-mer probes used in Affymetrix Mouse Gene Expresson Arrays were downloaded from the manufacturer's website [65]. For each version of these arrays the probe sequences were used to BLAT search the mm9 release of the mouse genome by individual chromosome. The BLAT search parameters were -minIdentity = 95 -tileSize = 11 -stepSize = 5 -repMatch = 1^10^. The genome co-ordinates of the top hit (with a 24/25 nt identity minimum cut-off) for each probe sequence were compared to the co-ordinates of the Repeatmasked regions of the mm9 release of mouse genome using a *Perl* script. Probes that overlapped a Repeatmasked region by at least 24 nt, and were in the appropriate orientation to recognize sense transcripts, were selected and annotated with the Repeatmasker class, family, and element corresponding to that genomic region. Tables containing the repetitive element probes for each Affymetrix array platform were imported into R. These annotation tables are available with the online version of this paper (Datasets S4, S5).

### Pre-processing of Affymetrix Microarray Gene Expression Data

Affymetrix Mouse Gene Expression data for *Ring1B^−/−^*, *Eed^−/−^*, *Ring1B^−/−^ Eed^−/−^*, *Hdac1^−/−^* and Dnmt TKO ES cells were downloaded from the NCBI GEO repository [38], accession numbers GSE19076 [26], GSE20177 [41] and GSE5583 [43]. Raw Affymetrix data were imported into the *affy* Bioconductor package [66] in R. Probe expression values were background-corrected using the robust multi-array average algorithm [67] in affy. Expression values for the perfect match probes were extracted from *affy*, log-transformed, then quantile-normalized. Summation across probesets was not performed so that the Affymetrix data could be analysed at the probe level. Probes that were expressed at more than the sample median level in at least half the arrays for one experimental condition in a dataset were considered to be present [68]. Absent probes were removed from the dataset to simplify the analysis. Some probe sequences in the Affymetrix Gene Expression platform are present in more than one probeset, and these redundant probes are present at multiple locations in the array. Therefore some 25-mer DNA sequences are represented by more than one probe in the Affymetrix datasets.

### Identification of Differentially Expressed Probes in Illumina and Affymetrix Microarray Data

For both Illumina and Affymetrix data, the R Bioconductor package *limma* [69] was used to identify probes that were expressed at different levels in experimental and control conditions by linear modeling. The Benjamini-Hochberg method was used to correct for multiple testing in *limma*, and adjusted p-values of ≤0.01 were considered to be statistically significant. Tables corresponding to all expressed probes in the experiment, and probes that statistically changed during the experiment, were repeat-annotated in R using the tables generated in sections 2.5 and 2.7. The resulting data were graphed using R. MA-plots show the log fold change in expression of each probe, plotted against the average expression of that probe in the dataset. Probability density functions for the microarray data were generated by kernel density estimation in R.

### Phylogenetic Analysis

Close relatives of *MMERVK10C* were found by using *MMERVK10C* as a template for Genewise [70] to predict pol and *pro* sequences in the Repbase database of repetitive DNA sequences [3]. Multiple protein alignment was performed using ClustalW [71], and phylogenetic trees were constructed using MEGA4 [72] to apply the neighbour-joining method [73]. Phylogenies were based on the proportion of amino acid sites at which sequences are different, with pairwise deletion to remove gaps in alignments as the need arises. The reliability of each interior branch of a given topology was assessed using the bootstrap interior branch test with 1000 bootstraps.

### Assembly of Repeatmasker Genomic Hits into Contigs

The co-ordinates of the Repeatmasked regions of the mm9 release of the mouse genome were downloaded from the UCSC genome browser [62], and regions Repeatmasked for *MMERVK10C-int* or *IAP-int* were extracted. The hits were ordered by their co-ordinates and adjacent hits that were in the same orientation on the same chromosome, were collinear on the consensus sequence, and were separated by less than the length of the consensus sequence were assembled into the same contig. *IAP*-int contigs that had *IAP* LTRs located within 50 bp of both ends of the contig were identified for further analysis. A similar approach was used to identify *RLTR10*-flanked *MMERVK10C* contigs, with *RLTR10* genomic loci greater than 250 bp included in the assembly.

### LTR Retrotransposon Enrichment in ChIP-seq Data

Hdac1 ES cell ChIP-seq and control ES cell whole cell extract datasets were downloaded from the GEO repository (accession number GSE27844). LTR retrotransposon enrichment was calculated using the Repeat Enrichment Estimator web application [25], and data for either all IAP LTR sequences, or all *RLTR45* LTR sequences, were combined.

## Supporting Information

Dataset S1 Tab-delimited text file containing complementary repeat probes in Illumina Mouse 6V1 Beadchips.
(TXT)

Dataset S2 Tab-delimited text file containing complementary repeat probes in Illumina Mouse 6V1.1 Beadchips.
(TXT)

Dataset S3 Tab-delimited text file containing complementary repeat probes in Illumina Mouse 6V2 Beadchips.
(TXT)

Dataset S4 Tab-delimited text file containing complementary repeat probes in Affymetrix Murine Genome U74Av2 GeneChips.
(TXT)

Dataset S5 Tab-delimited text file containing complementary repeat probes in Affymetrix Mouse430 2.0 GeneChips.
(TXT)

Figure S1 PDF showing a schematic diagram of Repeatmasker organization of murine repetitive elements into classes and families. The 1221 different consensus sequences for murine repetitive elements are categorized into 45 families within 16 classes by Repeatmasker. The organization of the repetitive elements most relevant for this study are shown schematically in the figure, and the number of consensus sequences belonging to each class and family are indicated. Examples of LTR retrotransposons belonging to each of the four main LTR retrotransposon families are also shown.
(PDF)

Figure S2 PDF showing the sequences of primers used for qRT-PCR in this study.
(PDF)
