# Supplementary figures and images for: Microarray Analysis of LTR Retrotransposon Silencing Identifies Hdac1 as a Regulator of Retrotransposon Expression in Mouse Embryonic Stem Cells
Source: PLoS Comput Biol. 2012 Apr 26;8(4):e1002486. doi: 10.1371/journal.pcbi.1002486 (PMC3343110; doi:10.1371/journal.pcbi.1002486)

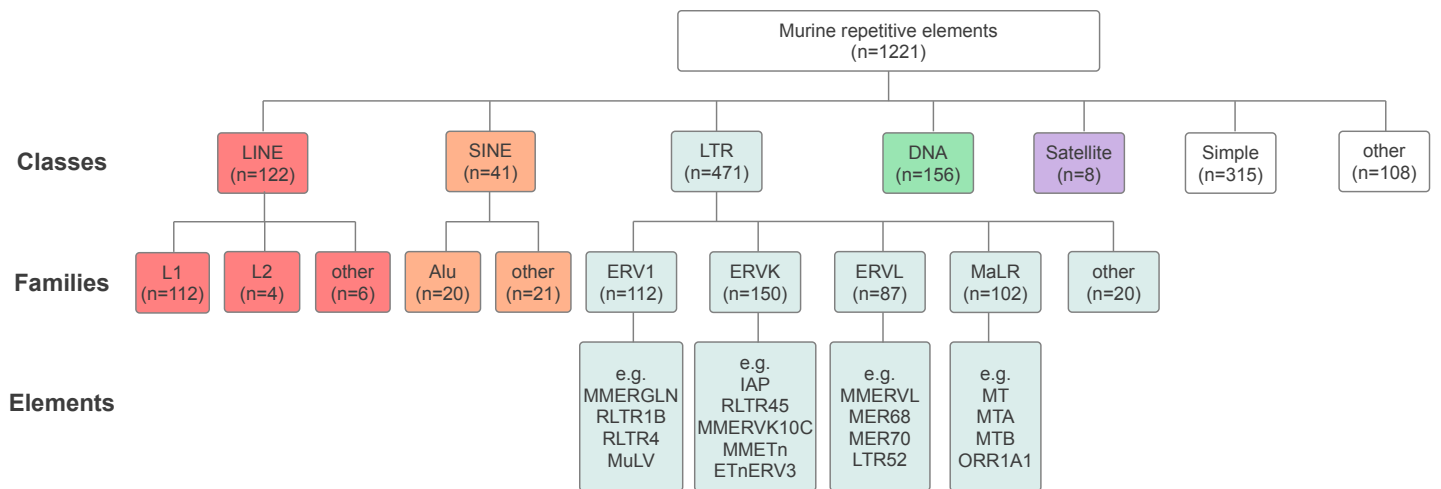

Supplement: Figure S1 — PDF showing a schematic diagram of Repeatmasker organization of murine repetitive elements into classes and families. The 1221 different consensus sequences for murine repetitive elements are categorized into 45 families within 16 classes by Repeatmasker. The organization of the repetitive elements most relevant for this study are shown schematically in the figure, and the number of consensus sequences belonging to each class and family are indicated. Examples of LTR retrotransposons belonging to each of the four main LTR retrotransposon families are also shown. (PDF) [file pcbi.1002486.s006.pdf]
