# Supplementary material for: Microarray Analysis of LTR Retrotransposon Silencing Identifies Hdac1 as a Regulator of Retrotransposon Expression in Mouse Embryonic Stem Cells
Source: PLoS Comput Biol. 2012 Apr 26;8(4):e1002486. doi: 10.1371/journal.pcbi.1002486 (PMC3343110; doi:10.1371/journal.pcbi.1002486)
Supplement: Figure S2 — PDF showing the sequences of primers used for qRT-PCR in this study. (PDF) [file pcbi.1002486.s007.pdf]

Gene / Repeat	Primer Sequences
β-Actin	5'-AGAGCTATGAGCTGCCTGACG-3'<br>5'-TGTGTTGGCATAGAGGTCTTTACG-3'
Gapdh	5'-ACCCAGAAGACTGTGGATGG-3'<br>5'-GGTCCTCAGTGTAGCCCAAG-3'
Sdmg1	5'-TCTGTGTTGAGATGCTGTTCG-3'<br>5'-AGGCTGGCGAGAAGTTATGA-3'
IAP	5'-GCACCCTCAAAGCCTATCTTA-3'<br>5'-TCCCTTGGTCAGTCTGGATTT-3'
IAP.int.1	5'-CAGAAGATTCTGGTCTGTGGTGTT-3'<br>5'-GAATTCATACAGTTGAATCCTTCT-3'
IAP.int.2	5'-AGCAGGTGAAGCCACTG-3'<br>5'-CTTGCCACACTTAGAGC-3'
IAP.chr10	5'-GTGCTCTGCCTTACAACTCG-3'<br>5'-AAGACGCAGCAAACCAGAAT-3'
LINE1 5'UTR	5'-GGAGTCTGCGTTCTGATGA-3'<br>5'-GGCGAAAGGCAAACGTAAGA-3'
LINE1 5'UTR.b	5'-GAAATTAGTCTGAACAGGTGAGAGG-3'<br>5'-TCCTCTGGTCCGGAAGGT-3'
LINE1 ORF2	5'-GGAGGGACATTTCATTCTCATC-3'<br>5'-GCTGCTCTTGTATTTGGAGCATAGA-3'
MERVL2a	5'-GCCAGAGAGGTGCGGCAGTGGGC-3'<br>5'-GGACCCGTGGATCCTGGCTGTGGGA-3'
ETnERV2	5'-ACAAATTCAGTATGGGCATC-3'<br>5'-GGGTACTGTTAAGACCCACA-3'
IAPEY3	5'-ACAGAGGAGGACAACTGCTC-3'<br>5'-AACCTTACACAGGCAAAAGC-3'
MMERVK10C gag	5'-CCTTTCCATGGTGTGATAGC-3'<br>5'-TGAATTTGTAGCTCGCATGA-3'
MMERVK10C pol	5'-GTGATCTTCCAGCATCATCC-3'<br>5'-GCCAAGGCATAATTGAGAGA-3'
MMERVK10C env.a	5'-TTTGTTTTGTCACCCCCACT-3'<br>5'-TTCCAGTCAGCAATGCAAAG-3'
MMERVK10C env.b	5'-CTTACCCTGCAAAAGGTGGA-3'<br>5'-TGGATGCCACACAACTCATT-3'
MMERVK10C env.c	5'-AACTGGTCGCAGGAGCTG-3'<br>5'-GGTAAAGTCTCCGAGGGTCA-3'
MMERVK10C env.d	5'-CCATATGCTGTGGACTGGTG-3'<br>5'-GCCGTTTTCAATGGCTAAAA-3'
MMERGLN	5'-CCTGGAGGATTTGACCTAGACA-3'<br>5'-CCCAGGTATACCCTTTTGTCC-3'
RLTR4.int.chr8	5'-CATACTTCTGCCCCAGCTAA-3'<br>5'-CAGTAATCGGTGGTGAGGTC-3'
RLTR4.int.1	5'-ACAAAGGCCTCCTCACTTCT-3'<br>5'-TGCCCTCATCTTCTGATAGC-3'
RLTR4.int.2	5'-TGGCCCATTCTGTATCAGTT-3'<br>5'-AGTTACGGTCTGTCCCATGA-3'
MuLV.int	5'-GGCAGCCATACATACAGACC-3'<br>5'-TGGTCTGCATAGAAACAGCA-3'
RLTR45	5-TGCTTTTCCGACATGGTAAT-3'<br>5'-AGTAACCCTGACCTGCTCCT-3'
